# Parallel Germline Infiltration of a Lentivirus in Two Malagasy Lemurs

**DOI:** 10.1371/journal.pgen.1000425

**Published:** 2009-03-20

**Authors:** Clément Gilbert, David G. Maxfield, Steven M. Goodman, Cédric Feschotte

**Affiliations:** 1 Department of Biology, University of Texas at Arlington, Arlington, Texas, United States of America; 2 Department of Zoology, Field Museum of Natural History, Chicago, Illinois, United States of America; 3 Vahatra, BP 3972, Antananarivo, Madagascar; Fred Hutchinson Cancer Research Center, United States of America

## Abstract

Retroviruses normally infect the somatic cells of their host and are transmitted horizontally, i.e., in an exogenous way. Occasionally, however, some retroviruses can also infect and integrate into the genome of germ cells, which may allow for their vertical inheritance and fixation in a given species; a process known as endogenization. Lentiviruses, a group of mammalian retroviruses that includes HIV, are known to infect primates, ruminants, horses, and cats. Unlike many other retroviruses, these viruses have not been demonstrably successful at germline infiltration. Here, we report on the discovery of endogenous lentiviral insertions in seven species of Malagasy lemurs from two different genera—*Cheirogaleus* and *Microcebus*. Combining molecular clock analyses and cross-species screening of orthologous insertions, we show that the presence of this endogenous lentivirus in six species of *Microcebus* is the result of one endogenization event that occurred about 4.2 million years ago. In addition, we demonstrate that this lentivirus independently infiltrated the germline of *Cheirogaleus* and that the two endogenization events occurred quasi-simultaneously. Using multiple proviral copies, we derive and characterize an apparently full length and intact consensus for this lentivirus. These results provide evidence that lentiviruses have repeatedly infiltrated the germline of prosimian species and that primates have been exposed to lentiviruses for a much longer time than what can be inferred based on sequence comparison of circulating lentiviruses. The study sets the stage for an unprecedented opportunity to reconstruct an ancestral primate lentivirus and thereby advance our knowledge of host–virus interactions.

## Author Summary

Retroviruses are RNA viruses that are reverse transcribed into DNA and inserted into the host's genome. Though this process happens most frequently in somatic cells (e.g., immune cells for HIV), retroviruses can occasionally be integrated in the genome of the host's germ cells. Such viral insertions may thus be transmitted vertically from parent to offspring, leading to the formation of “endogenous retroviruses.” A substantial fraction of mammalian genomes (about 8% in humans) corresponds to remnants of endogenous retroviruses integrated throughout evolution, providing a fossil record of past viral invasions and important clues on the history of modern retroviruses. In this study, we demonstrate that an endogenous retrovirus related to HIV and other lentiviruses was endogenized independently and quasi-simultaneously in two lineages of Malagasy lemurs around 4.2 million years ago. These are the first endogenous lentiviruses discovered in primates. Based on sequences collected from different lemur species, we reconstructed an apparently intact and complete sequence for this ancestral prosimian lentivirus, which will allow functional analysis and advance our understanding of the biology and origin of lentiviruses, including HIV. Furthermore, our study indicates that lentiviruses may still be circulating in lemurs and that a systematic screening of Malagasy mammals could further our knowledge on the past and present diversity of lentiviruses.

## Introduction

Lentiviruses are mammalian retroviruses known to infect cattle, cats, horses, sheep, and primates. They are the focus of intense study due to their causative association with AIDS in human. Although our knowledge on the origin and early evolution of HIV has grown exponentially over the past few years [1],[2], much remains unresolved about the deeper relationships between primate and non-primate lentiviruses, the origin of lentiviruses, and their mode of structural evolution over long periods of evolutionary time. This is because these viruses evolve extremely rapidly [3], in a conflicting relationship with their hosts [4], and while their high mutation rate provides a wealth of information documenting their recent history, it also quickly erases evidence of their deeper ancestry.

The lifecycle of retroviruses is atypical compared to other viruses in that after appropriate receptor recognition and entry in a specific cell type, their RNA genome is reverse transcribed into double-stranded DNA and integrated into the host genome as a provirus [5]. Occasionally this process can take place in the host germline, and the integrated copy, also called endogenous retrovirus (ERV), may be transmitted vertically from parent to offspring and reach fixation in the host population. As such, ERVs constitute a “fossil record” of past viral infections that potentially provide an alternative way of gaining insights into the deep evolutionary history of present day exogenous retroviruses [6].

Although many ERVs have been characterized in mammals (e.g., 8% of the human genome), apparently very few derive from lentiviruses. Two reasons have traditionally been put forward to explain their absence in mammalian genomes: (i) they are of relatively recent evolutionary origin and endogenization has not yet commonly occurred, and/or (ii) they were not able to enter germ cells because of a very specific cell tropism [7],[8]. Recently however, an endogenous lentivirus, called RELIK, has been identified in the genome of rabbits and hares (Lagomorpha), whose germline integration was dated at least 12 millions years (my) old [9]–[11]. This discovery not only showed that lentiviruses were able to infiltrate mammalian germlines, but also demonstrated that this group of viruses is probably much older than what could previously be inferred based on sequence comparison of extant exogenous lentiviruses.

Even more recently, Gifford et al. [12] described the remnants of an endogenous lentivirus in the genome of the prosimian primate *Microcebus murinus*. This virus, called pSIVgml for “gray mouse lemur prosimian immunodeficiency virus”, represents the first example of a primate endogenous lentivirus. Here we report on our independent discovery and characterization of pSIVgml and of a second, closely related endogenous prosimian lentivirus, pSIVfdl, which independently colonized the genome of the fat-tailed dwarf lemur *Cheirogaleus medius*. Our analyses of these defective proviral sequences corroborate and expand the findings of Gifford et al. [12] and allow us to reconstruct an apparently full-length and intact pSIV consensus sequence that provides new insights into the evolutionary history of lentiviruses and should permit functional analysis of an ancestral primate lentivirus.

## Results/Discussion

### Discovery of an Endogenous Lentivirus in the Gray Mouse Lemur Genome

Homology based searches (tBLASTn) of whole genome shotgun (WGS) sequences using the rabbit endogenous lentivirus (RELIK) consensus sequence [9] as a query yielded highly significant hits in the *gag* and *pol* domains to two contigs from the gray mouse lemur (*Microcebus murinus*) genome sequencing project (Table 1). Further BLASTn searches on the *M. murinus* WGS sequences (1.93× June 2007 release) using the *M. murinus pol*-containing contig (ABDC01505939) as a query yielded ten other contigs containing a fragment highly similar to the region situated upstream of the *pol* domain, i.e., the presumed long terminal repeat (LTR). Five of these fragments (413–423 bp in length) are flanked by short direct repeats akin to target site duplications (TSD, Table 1) and therefore likely correspond to solo LTRs resulting from intra-element recombination [13]. Four other hits correspond to LTRs truncated due to sequencing or assembly gap, and one corresponds to a 3′ full-length LTR flanking an *env* domain also truncated due to a gap.

**Table 1:** Summary of all the fragments of pSIVgml found in the whole genome shotgun sequence database of the gray mouse lemur, *Microcebus murinus* (1.93× coverage).

Accession number	Size of contig	Position of hit in contig	Region of pSIVgml	TSD
ABDC01454291	2278	1–2278	truncated pol	-
ABDC01505939(-)	2008	1–2008	truncated 5′ LTR, gag and truncated pol	-
ABDC01306160*	2572	1–874	truncated env+LTR	ATTAT
ABDC01159233(-)	7096	3280–3698	solo LTR	AAAGG
ABDC01341005	1986	95–507	solo LTR	AATTA
ABDC01361523(-)	3543	2823–3241	solo LTR	CTTCC
ABDC01457045	1834	1344–1766	solo LTR	ATTAT
ABDC01486581	2666	321–740	solo LTR	GTTCT
ABDC01454290	2698	2315–2698	truncated 5′ LTR	CCCCA
ABDC01501173(-)	2760	2435–2760	truncated solo LTR	ACTTC
ABDC01638355	955	2–120	truncated solo LTR	GGTAG
ABDC01223095	2841	2728–2841	truncated solo LTR	TGTGA

(-) indicates when the fragment is on the minus strand of the contig. *this contig corresponds to a missassembly of two trace reads (1556822362 and 1562873896) (see text and Figure S1). Searches of the trace archives conducted by (Gifford et al., 2008) yielded one more solo-LTR (trace reads 1560845536 and 1550208878) not identified in the WGS database.

These results are broadly consistent with Gifford et al. [12] who undertook an approach similar to ours, except that these authors also searched the trace archives database and found an additional solo-LTR that we did not detect in the WGS database (Table 1). Below we confirm that these proviral fragments correspond to an endogenous lentivirus identical to the one described in [12] and thus we adopt the nomenclature introduced by these authors who named this lentivirus pSIVgml for gray mouse lemur prosimian immunodeficiency virus.

### Copy Number and Taxonomic Distribution

**Figure 1:**
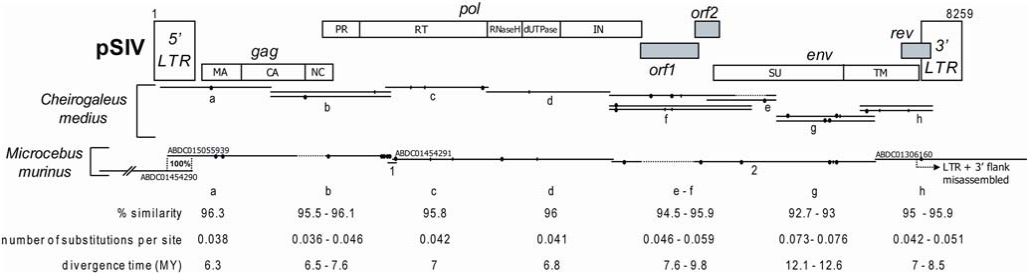
Map of the consensus pSIV reconstructed in this study based on one full-length copy of pSIVgml (*Microcebus murinus*) and between one and three copies of pSIVfdl (*Cheirogaleus medius*) (the alignment is provided in Dataset S1). The LTR fragments contained in the ABDC01505939 and ABDC01454290 contigs correspond to the same full length pSIV copy (see Figure S1). The contig ABDC01306160 results from a misassembly between a trace read containing a solo-LTR and a trace read containing the 3′ terminus of the *env* of the full-length pSIVgml and a fragment of the 3′ LTR (see Figure S1). The different domains of pSIV were identified by comparison with the HIV1-HXB2 reference sequence [40] (see Figure S4 for a more precise map). Closed circles: non-sense frameshifts. Vertical bars: in frame stop codons. Dash lines: missing fragments. The range of pairwise similarity, number of substitutions per site and inferred divergence times between pSIVgml and pSIVfdl sequences are indicated.

The coverage of the gray mouse lemur genome is low (1.93×) and its assembly still very fragmentary, implying that any estimate of pSIVgml copy number based only on database mining will be tentative at best. Two of the pSIVgml LTRs in the *M. murinus* WGS were associated to internal coding sequences (contig ABDC01454290/ ABDC01505939 and contig ABDC01306160) suggesting that they represent the 5′ and 3′ LTRs of seemingly full-length proviruses. Since these LTRs were not flanked by the same TSD (CCCCA *vs.* ATTAT) (Table 1, Figure 1), Gifford et al. [12] concluded that at least two distinct full-length proviral insertions must exist in the genome of *M. murinus*. Based on this observation and assuming that the amount of sequence deposited in the WGS database corresponds roughly to 30% of the complete *Microcebus* genome, the authors estimated that there may be up to six full length copies of pSIVgml [12].

**Figure 2:**
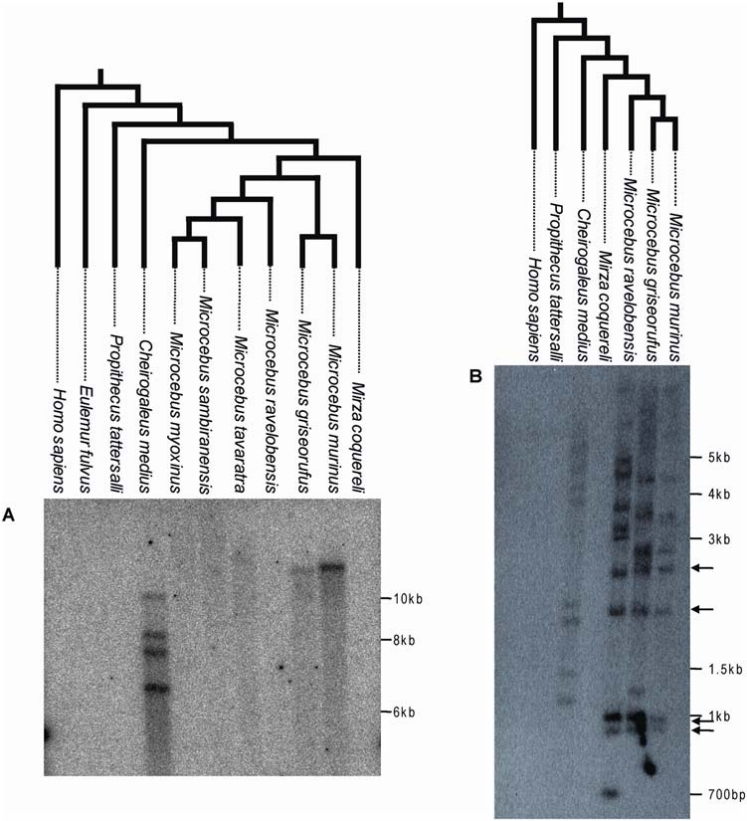
Southern blot of digested genomic DNA of various Malagasy lemurs and human using a ∼1 kb probe corresponding to a fragment of pSIVgml *env* (A) or a ∼300 bp probe corresponding to a fragment of the pSIV LTR (B). Arrows highlight bands of the same size shared by *Microcebus murinus*, *M. griseorufus* and *M. ravelobensis* but not *Cheirogaleus medius*. These bands likely correspond to solo-LTRs located at orthologous position in the three *Microcebus* species. The trees on top of each blot depict the phylogenetic relationships of the species according to [23],[41]. See Table S4 for the voucher specimen numbers of the lemur tissue samples used in this study. A picture of the ethidium bromide stained gels used to prepare the blots is shown in Figure S2. The absence of pSIV in *Mirza* was confirmed by PCR using different sets of primers (Figure S3).

As a more direct approach to estimate the copy number of pSIVgml and to screen for the possible presence of related endogenous lentiviruses in related prosimian species, we performed Southern hybridizations of digested total genomic DNA from *M. murinus*, nine other species of Malagasy lemurs and *Homo sapiens* as a negative control. A ∼1-kb probe corresponding to a fragment of the pSIVgml *env* gene revealed only one band in *M. murinus* (Figure 2A), which was inconsistent with the copy number estimate based on database mining (i.e. between two and six full length copies) [12]. In order to identify the origin of this discrepancy, we sought to validate the WGS draft assembly of *M. murinus* using PCR with primers anchored in the sequence reads used for the initial assembly. We were able to confirm that contigs ABDC01454290 and ABDC01505939 can be assembled into a single contig containing a 5′ LTR adjacent to a *gag* and partial *pol* genes, indicating that this locus is likely to correspond to a full-length proviral insertion (Figure S1). We were unable to recover any PCR products using a forward primer located in the *env* region and a reverse primer located in the assigned 3′ flanking region of contig ABDC01306160 (Figure S1). We also observed that the two trace reads (1556822362 and 1562873896) used to assemble contig ABDC01306160 overlap within the LTR, which suggests that the *env* region could have been misassembled to an illegitimate 3′ LTR. We suspected that this *env* gene was in fact associated with the full-length proviral insertion aforementioned and characterized by the CCCCA TSD (Figure S1). This was confirmed by amplifying a PCR product spanning the *env* region, 3′ LTR and flanking genomic DNA with the CCCCA 3′ TSD. Sequencing of this PCR product revealed 100% identity with the *env* gene in contig ABDC01306160 (Figure S1), suggesting that indeed we had connected the single *env* gene present in the genome to its legitimate 3′ LTR. Together with the Southern results, these data point to the presence of a single full-length pSIVgml provirus in the *M. murinus* genome and that contig ABDC01306160 is the result of a misassembly in the draft genome sequence.

The Southern analysis showed that pSIV is not restricted to the gray mouse lemur but is also present in low copy number in several additional Malagasy lemurs. The *env* probe revealed one band in *M. griseorufus*, four bands in *Cheirogaleus medius* and no bands in the other lemur species examined (*M. ravelobensis*, *M. myoxinus*, *M. tavaratra*, *M. sambiranensis*, *Mirza coquereli*, *Propithecus tattersalli*, *Eulemur fulvus rufus*) (Figure 2A; see also Figure S2A, Figure S3). A second probe corresponding to a ∼300-bp LTR fragment hybridized with 8 to 11 genomic fragments in *M. griseorufus*, *M. murinus*, *M. ravelobensis*, and *C. medius* but yielded no hybridization in the three other lemur species (Figure 2B, Figure S2B). Assuming no intra-element restriction site polymorphism, these results suggest that there are at least four potentially full-length (i.e., insertions including some coding region, as opposed to solo LTR) pSIV proviruses in *Cheirogaleus* and only one in *M. murinus* and *M. griseorufus*. The genomes of *M. ravelobensis*, *M. myoxinus*, *M. tavaratra* and *M. sambiranensis* seem to harbor only solo LTRs, and pSIV is absent from *Mirza*, *Propithecus*, and *Eulemur*.

### Reconstruction of a pSIV Consensus

Using PCR primers (Table S1) designed upon the pSIVgml-containing contigs, we sequenced the missing fragments of pSIVgml in *Microcebus murinus* and multiple clones covering what appears to represent a full-length pSIV in *Cheirogaleus medius* (Figure 1; Dataset S1) that we named pSIVfdl for “fat-tailed dwarf lemur prosimian immunodeficiency virus”, following the nomenclature introduced by Gifford et al. [12]. The pSIV sequences obtained in both species of these two genera contain a substantial amount of frameshifts, stop codons, and some large deletions, indicating that the pSIV insertions are defective and relatively ancient. Sequence similarity between pSIVgml and pSIVfdl is remarkably high (93–96%) compared to the genetic diversity observed within HIV-1 subtypes (80–85%) [14], suggesting that the viruses endogenized in the two lemur species were nearly identical. We therefore decided to use all sequences from both species to reconstruct a single pSIV consensus (Figure 1 and S4).

Though overall the structure of our pSIV consensus is largely consistent with the pSIVgml sequence reported by Gifford et al. [12], the inclusion of additional pSIVfdl proviral copies (from *Cheirogaleus*) allowed us to fill several gaps that are apparent in pSIVgml. The revised pSIV consensus is now free of stop codons and non-sense frameshifts since none of the mutations was shared between pSIVgml and the various pSIVfdl copies. In addition, the fragment including the 3′ end of the capsid, nucleocapsid and the 5′ end of the protease domains that is missing in the pSIVgml sequence (Figure 1, this study; Figure 1 in [12]) was present in the two pSIVfdl clones overlapping the *gag* and *pol* genes (clones d; Figure 1). Close inspection of the complete *gag*-*pol* junction revealed that the translation of the *pol* gene is most likely regulated via −1 frameshifting (Figure S4), which is characteristic of most known retroviruses [5].

We confirm the presence of a putative *rev* accessory gene overlapping with the 3′ end of the *env* open reading frame (ORF), but the two different copies of pSIVfdl included in our analysis do not contain the stop codon separating the *rev* gene from the putative terminal small ORF identified in [12]. Consequently, the putative *rev* gene characterized here encompasses the sequence corresponding to this 3′ putative ORF and terminates with a motif rich in leucine residues, characteristic of the nuclear export signals found in *rev* and other nuclear transporters [15],[16].

The most significant difference between the pSIV consensus and the previously reported pSIVgml sequence [12] lies in the region situated between *pol* and *env*. The three different pSIVfdl clones covering this region (clones f1, f2 and e1) all contain a 511-bp region that is apparently deleted in pSIVgml (Figure 1). Analysis of the complete *pol*-*env* intervening region revealed two small overlapping ORFs that we named *orf1* and *orf2*. The 5′ end of *orf1* slightly overlaps with the end of *pol* while its 3′ end comprises the first 22 amino acid (aa) of the putative *vif* identified in [12]. The sequence for *orf2* largely overlaps with the initially characterized *vif* while the start of *env* corresponds to the start of the putative *tat* accessory gene proposed in [12]. We could not detect any significant similarity between *orf1* (199 aa) and *orf2* (83 aa) and any known lentiviral accessory gene, but we note that they are located at a comparable genomic position than *vif* and *tat*, i.e., between *pol* and *env*, and the predicted proteins are very similar in size to those encoded by these accessory genes in other primate lentiviruses (*vif* is 192 aa and *tat* is 86 aa in HIV1-HXB2). Thus, it is possible that these pSIV ORFs encode *vif* and *tat* homologs.

**Figure 3:**
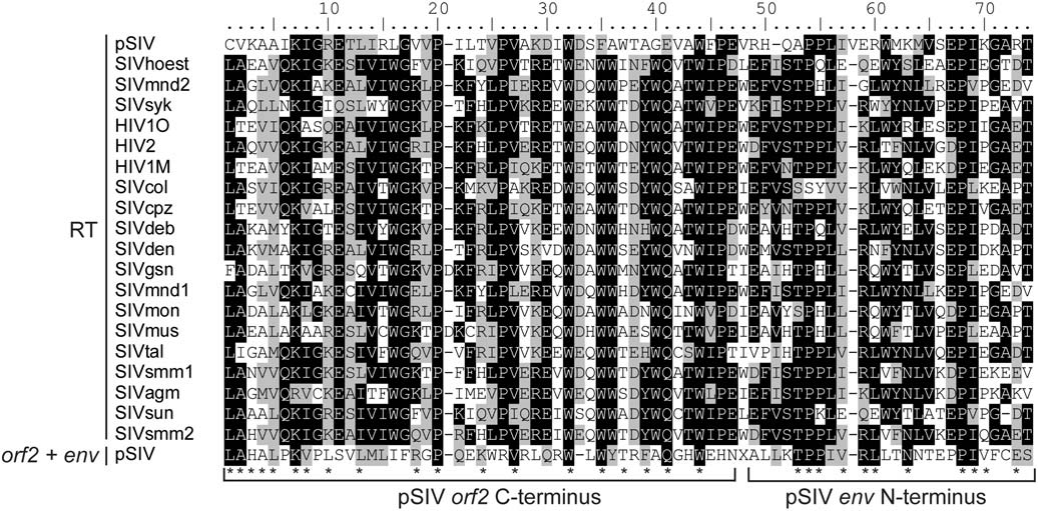
Alignment between a 71 aa region of the RT domain of the primate lentiviruses and the *orf2* (46 aa)−*env* (25 aa) junction of pSIV. Shading of the different positions represents the level of sequence conservation using the BLOSUM 62 amino acid substitution matrix in BioEdit (Hall, 2004). In addition, each asterisk indicates positions where pSIV amino acids are shared with at least one other primate lentivirus sequence. Accession numbers of the sequences are listed in Table S2.

Interestingly, tBLASTn searches using the pSIV consensus as query yielded weak but significant similarity (e-value = 0.041) with the C-terminus of the reverse transcriptase encoded by primate lentiviruses in a region of pSIV including 46 aa of the *orf2* C-terminus and 25 aa of the *env* N-terminus (Figure 3). This is reminiscent of the situation reported for HIV-2, where the *vpx* gene (found only in HIV-2) shows significant similarity to the *vpr* gene (found in all simian lentiviruses) [17]. Based on this observation and on phylogenetic analyses of different simian lentiviruses, it has been proposed that *vpx* originated through non-homologous recombination between one strain of SIV and an early ancestor of HIV-2 [18],[19]. Likewise, non-homologous recombination at the RNA level via template switching between two ancient lentiviral genomes may have resulted in the transfer of part of the RT sequence between the *pol* and *env* domains of a pSIV ancestor, giving rise to a large portion of *orf2*, the putative *tat* homolog. This finding supports the potential key role of the highly error-prone lentiviral reverse transcriptase in generating new viral variants through reshuffling of their genomes [20].

### Phylogenetic Analyses

**Figure 4:**
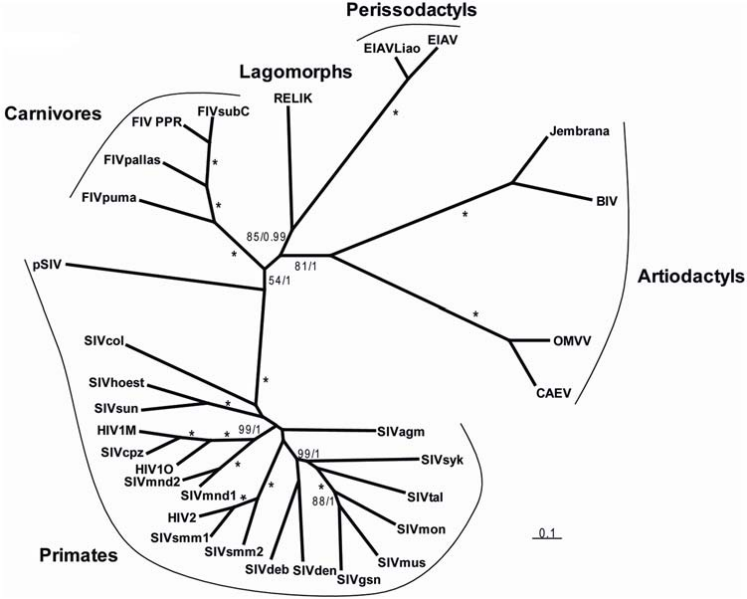
Unrooted tree of lentiviruses obtained after phylogenetic analysis of an alignment including ∼2350 nucleotides of the *gag*-*pol* region. Numbers associated to internal branches correspond to Bayesian posterior probabilities/bootstrap ML values. Asterisks indicate when an internal branch is supported by posterior probability = 1/bootstrap = 100. Accession numbers of the sequences are listed in Table S2. The alignment used for the analyses is provided in Dataset S2.

In order to formally assess the phylogenetic relationships between pSIV and other retroviruses, we performed Bayesian and Maximum Likelihood (ML) phylogenetic analyses of the well-conserved reverse transcriptase (RT) domain. Both methods unequivocally support the grouping of pSIV within the lentivirus clade (Figure S5). Furthermore, as the RT alone does not provide any phylogenetic resolution between the different genera of lentiviruses, we also conducted Bayesian and ML analyses of the *pol* and *gag* domains extracted from a diverse set of lentiviruses. Separate analysis of the two domains did not reveal any obvious recombination event, i.e., the *gag* tree was not incongruent with the *pol* tree (not shown). In agreement with Gifford et al. [12], the Bayesian analysis combining *gag* and *pol* provided strong support for a potential sister relationship between pSIV and other primate lentiviruses, but this grouping is somewhat equivocal since the support was much lower in the ML analysis (Figure 4). Regardless, we believe that pSIV is sufficiently distant from the other known lentiviruses to be considered as a distinct lentiviral species.

### How Many pSIV Germline Infiltrations?

**Figure 5:**
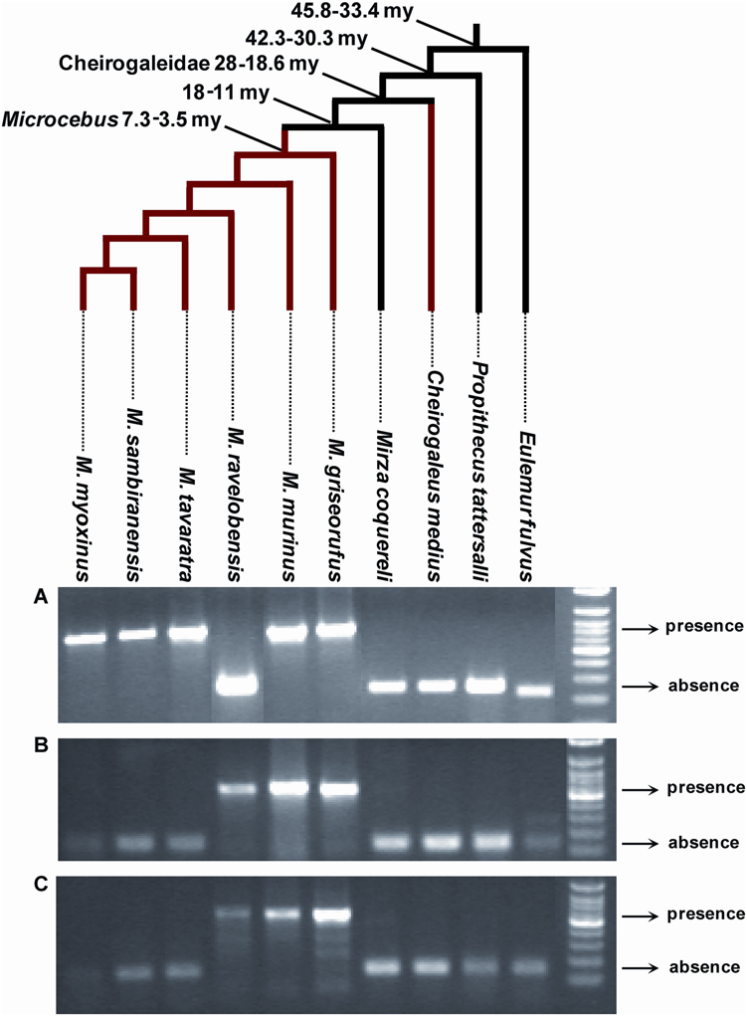
PCR screening for presence/absence of orthologous solo LTR in various species of Malagasy lemurs. For each locus, the larger PCR product indicates presence of the LTR, the smaller product indicates absence. (A) Primers (6160F: 5′-CAG CAK TTT CAT CAG CAA TTT G; 6160R: 5′-GCA AGC TGT GMC ACA TTT ATT BGC) were designed on the regions flanking the solo LTR in contig ABDC01306160. The expected size was ∼670 bp for presence and ∼250 bp for absence. (B) Primers (9233F: 5′-ATC TRT AGT CAA ATC CTG GG; 9233R: 5′-TAA TAC TCA CAA AAA CYT TAC C) were designed on the regions flanking the solo LTR in contig ABDC01159233. The expected size was ∼550 bp for presence and ∼130 bp for absence. (C) Primers (61523F: 5′-AAA TGA GTT TTG TTG CTC TRT YTC; 61523R: 5′-ATG TTR CTT TGG GTA GMT TG) were designed on the regions flanking the solo LTR in contig ABDC01361523. The expected size was ∼585 bp for presence and ∼165 bp for absence. The genus *Eulemur* and *Propithecus* belong to the family Lemuridae and Indriidae respectively. All the other species (genera *Cheirogaleus*, *Microcebus* and *Mirza*) belong to the family Cheirogaleidae. The tree depicts the phylogenetic relationships of the species and their divergence times according to [23],[41]. See Table S4 for the voucher specimen numbers of the lemur samples used in this study.

The Malgasy lemurs form a monophyletic group composed of four families (Cheirogaleidae (∼21 spp), Indriidae (11 spp), Lemuridae (19 spp) and Lepilemuridae (8 spp)) [21] that is thought to have colonized Madagascar only once, between 60 and 50 my ago, most likely by rafting across the Mozambique Channel from East Africa [22]. As within the Cheirogaleidae family *Microcebus* is more closely related to *Mirza* than to *Cheirogaleus* [23] (Figure 2, 5), the presence of pSIV insertions in *Microcebus* and *Cheirogaleus* but not in *Mirza* (Figure 2, S3) implies that pSIV either infiltrated the germline of the ancestor of the three genera and was subsequently lost in *Mirza* or alternatively, that it independently colonized the germline of the *Cheirogaleus* and *Microcebus* lineages. The first hypothesis would imply that the pSIV insertions are between ∼38 million years (my) (oldest date for the split between the clade grouping *Microcebus*, *Cheirogaleus* and *Mirza* and its sister taxa *Lepilemur*) and ∼19 my old (youngest date for the split between *Cheirogaleus* and the clade grouping *Microcebus* and *Mirza*) [23].

Under the single germline infiltration hypothesis, the total genetic distance between the different pSIVfdl and pSIVgml copies should correspond to the mutations accumulated on both *Microcebus* and *Cheirogaleus* lineages under the neutral substitution rates of these species. These genetic distances vary between 0.038 in *gag* and 0.076 substitutions per site in *env* (average = 0.05) (Figure 1). Using the neutral substitution rate previously estimated for bushbaby (*Otolemur garnetti*), an African prosimian, (3×10^−9^ substitutions per site per year) [24], we can infer an approximate insertion time ranging from 6.3 to 12.6 my (average = 8.4 my), i.e., significantly younger than the split of *Cheirogaleus* and *Microcebus* (19–35 my). Therefore, the level of divergence between pSIVfdl and pSIVgml does not seem consistent with a single germline infiltration that would have occurred in the common ancestor of these lemurs and rather indicates that pSIV independently infiltrated the germline of *Microcebus* and *Cheirogaleus* after these two genera diverged from each other.

**Figure 6:**
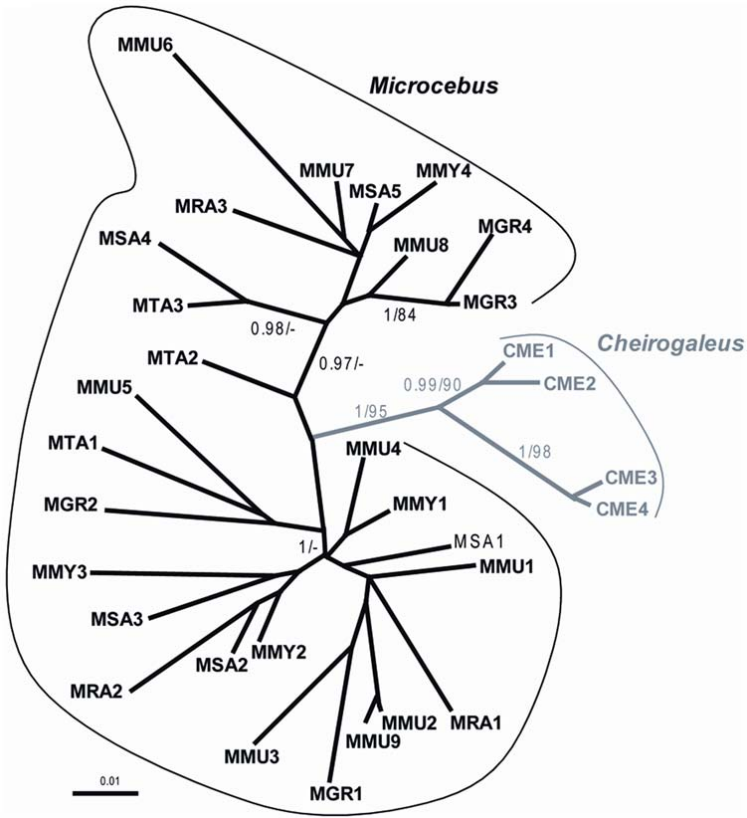
Unrooted tree of several LTRs (9<n<3) obtained in each of the following species of Malagasy lemurs. MMU: *Microcebus murinus*, MRA: *M. ravelobensis*, MTA: *M. tavaratra*, MSA: *M. sambiranensis*, MMY: *M. myoxinus*, MGR: *M. griseorufus*, CME: *Cheirogaleus medius*. See Table S4 for the voucher specimen numbers of the lemur samples used in this study. Numbers associated to internal branched correspond to Bayesian posterior probabilities ≥0.95/bootstrap ML values ≥80. The alignment used for the analyses is provided in Dataset S3.

Also consistent with the hypothesis of two independent germline infiltrations, the Southern blot (Figure 2) and PCR screening (Figure 5) of pSIV insertions in six *Microcebus* species and *Cheirogaleus* did not reveal any shared orthologous insertion between *Microcebus* and *Cheirogaleus*, as would be expected under the single ancestral germline infiltration model. In addition, sequencing and phylogenetic analysis of multiple pSIV LTRs from the different species of *Microcebus* and from *C. medius* yielded two distinct monophyletic clades that correspond to the two lemur genera (Figure 6). This shows that pSIVfdl and pSIVgml most likely derive from two closely related but distinct circulating lentiviruses, although the possibility of a gene conversion effect that would have homogenized the different LTRs in both species cannot be excluded [25].

Given the low number of pSIV proviruses, the lack of coding sequence for most of them (solo LTRs) and the high level of similarity between the pSIVfdl copies, we did not attempt to identify the mechanism(s) that produced multiple copies in the different lemur genomes. They could result from repeated germline insertions of the same or very similar circulating lentiviruses, intragenomic retrotransposition events, reinfection by an endogenized copy, or a mix of these mechanisms. For simplicity, we therefore refer to all insertions in each species using the term “germline infiltration” but we acknowledge that each insertion may or may not correspond to a different endogenization event, i.e, the integration of one exogenous virus in the germline followed by its vertical transmission to offspring and fixation in the species.

### How Old Are the Two pSIVs Germline Infiltrations?

In order to estimate the time of the pSIVgml insertions in the *Microcebus* genome, we sequenced four orthologous solo LTRs shared by different *Microcebus* spp. (see Figure 5). Genetic distances between the most divergent species was between 0.015 and 0.038 substitutions per site (average = 0.025) (Table S3), which corresponds to an approximate insertion time of between 2.5 and 6.2 my (average = 4.2 my), again seemingly incompatible with a single germline infiltration event predating the *Cheirogaleus*/*Microcebus* split ∼19–38 my ago. Interestingly, *M. murinus* shares at least one orthologous insertion with each of the five other *Microcebus* species (Figure 5), suggesting that the germline infiltration of pSIVgml in the *Microcebus* genus occurred before the speciation of the extant taxa, i.e., between 3.5 and 7.3 my ago (Figure 5) [23], which is consistent with our molecular clock estimates. We note, however, that none of the orthologous pSIVgml insertions examined was shared by all *Microcebus* species. The most likely explanation for this pattern is that each insertion was fixed or eliminated after these species diverged from each other through idiosyncratic lineage sorting of ancestral polymorphism, a phenomenon well documented in *Microcebus* [26].

Our dating of pSIV germline integrations (2.5 and 6.2 my; average = 4.2 my) is older than the one inferred by Gifford et al. [12] (1.9–3.8 my). These authors relied solely on a comparison of two LTRs that they interpreted as an allelic polymorphism for a full-length pSIVgml and its solo LTR remaining after recombinogenic deletion of the rest of the provirus. However, a closer inspection of the raw sequence reads used for WGS assembly reveals that this apparent polymorphism is an artifact resulting from an assembly error, a common occurrence in low-coverage draft genome sequences. We experimentally confirmed that these LTRs actually originate from two different loci erroneously associated due to the genome misassembly (as described above and in Figure S1). Our dating method, which combines sequence divergence comparisons and cross-species analysis of orthologous insertions, provides a more reliable estimate of the age of pSIVgml germline infiltration.

Because pSIV apparently colonized at least twice independently the germline of lemurs, the total genetic distance between pSIVfdl and pSIVgml copies is expected to be the sum of (i) the mutations accumulated under the host neutral substitution rate on both *Microcebus* and *Cheirogaleus* branches since the time of each germline infiltration and (ii) the mutations accumulated under the viral substitution rate during the time separating the two germline infiltrations. As shown above, the average divergence between pSIVfdl and pSIVgml is 0.05 substitutions per site. We have also calculated the number of substitutions that occurred on pSIVgml since it integrated in the *Microcebus* germline, which is half of the orthologous LTR divergence, i.e., 0.025/2 = 0.0125 substitution per site. The cumulative number of substitutions that occurred on pSIV under the viral mutation rate and under the *Cheirogaleus* neutral substitution rate since germline infiltration is therefore approximately 0.05−0.0125 = 0.0375 substitutions per site. Lentiviral substitution rates differ from mammalian neutral substitution rates by 6 orders of magnitudes. The HIV substitution rate has been estimated to vary between 10×10^−3^ (synonymous substitutions) and 2×10^−3^ substitutions per site per year (non synonymous substitutions in *gag-pol*) [27]. Remarkably, under these rates, 0.0375 substitutions per site (as observed in pSIVfdl) are generated in only 3.75–18.75 years. Given the large difference between viral and mammalian neutral substitution rates, it is unlikely that any of the approximations made above would change this value by more than one or two orders of magnitude. This indicates that the time window separating the two germline infiltrations of pSIV was extremely narrow and thus these events must have occurred quasi simultaneously on an evolutionary time scale.

### Conclusions

In this study, we have confirmed the presence of an endogenous lentivirus in the genome of the Malagasy prosimian *Microcebus murinus* and its relatively close phylogenetic relationship with modern simian lentiviruses, as reported recently [12]. Given that Madagascar has been isolated from Africa for 160 million years [28], the presence of a lentivirus on this island raises several intriguing questions concerning the time, mode, and direction of the transfer of pSIV between Africa and Madagascar (see Gifford et al. [12] for a comprehensive discussion on this issue).

In addition, we have demonstrated that pSIV is also present in low copy numbers in the genome of several other species of *Microcebus* and in another Malagasy prosimian, *Cheirogaleus medius*. While the various pSIVgml insertions in *Microcebus* species are most likely the result of a single germline infiltration that occurred around 4.2 my ago before the split of the *Microcebus* genus, those detected in *Cheirogaleus* most likely stem from a second, independent germline infiltration, that occurred concomitantly to the one in *Microcebus*. These two synchronous lentiviral colonizations of the germline of two non-sister lemur genera are striking given the paucity of hitherto characterized endogenous lentiviruses. It is possible that they have been facilitated either by a broader cell tropism of pSIV (or at least of the particular variants of pSIV that led to endogenization) compared to most other lentiviruses, or that the germ cells of lemurs are particularly prone to lentiviral endogenization. In addition, the present geographic distributions of *Cheirogaleus* and *Microcebus* species widely overlap on Madagascar [29],[30]. Sympatry of the two genera, if already occurring at the time of pSIV endogenizations, may have also facilitated the horizontal transfer of pSIV between these lemurs. Although one study provides evidence of SIV antigens in the Malagasy ring tailed lemurs (diverged from the ancestor of *Microcebus* and *Cheirogaleus* between 45.8−33.4 my ago) based on western blot analysis [31], there is no direct evidence of circulating lentiviruses in prosimian primates. A systematic screening of the native Malagasy mammalian fauna for the presence of endogenous and/or exogenous lentiviruses might help us further our understanding of the origin and spread of pSIV and lentiviruses in general.

Finally, the inclusion of multiple copies of pSIVs allowed us to fill the different gaps that are apparent in the pSIVgml sequence, and to infer an apparently intact pSIV consensus suitable for experimental reconstruction and functional analysis. In this respect, it is noteworthy that our pSIV consensus contains a complete capsid domain and *pol-env* intervening region, with the later potentially encoding an accessory gene situated in the typical location and of the same size as *vif*. The capsid domain and *vif* accessory gene of HIV are known to interact respectively with TRIM5alpha [32] and APOBEC3 [33], two mammalian protein families involved in the restriction of lentiviruses and other retroviruses in their host. The identification of these two components in pSIV may allow testing of their interactions with TRIM5alpha and APOBEC3 proteins, which could further our understanding of the impact of these defense systems in shaping the evolution of lentiviruses.

## Materials and Methods

### PCR/Cloning/Sequencing

The PCR primers designed to amplify pSIV fragments in *Microcebus* and *Cheirogaleus* are listed in Table S1. Those used for the screening of presence/absence of orthologous solo-LTRs in the various species of lemurs and for testing the validity of contigs containing pSIV fragments are given in the caption of Figure S1 and 5. Standard PCR conditions were: 2 min at 94°C; 30 cycles of 1 min at 94°C, 30 s at 48–62°C, and 30 s–2 min at 72°C. PCR mix was: Buffer (5×), 5 ul; MgCl2 (25 mM), 2 ul; dNTP (10 mM), 0.5 ul; Primer 1 (10 uM), 1 ul; Primer 2 (10 uM), 1 ul; Taq (GoTaq, Promega), 1.25 U; DNA, 30–100 ng; and H2O up to 25 ul. PCR products were cloned into the pCR2.1-TOPO cloning vector (Invitrogen) and 4–6 randomly selected clones were sequenced on an ABI 3130XL sequencer. All sequences have been submitted to Genbank (Accession numbers: FJ707322–FJ707359).

### Southern Blot

Genomic Southern blots were prepared by digesting completely ∼5 µg of total genomic DNA from *Microcebus murinus*, *M. griseorufus*, *M. ravelobensis*, *Mirza coquereli*, *Cheirogaleus medius*, *Propithecus tattersalli*, *Eulemur fulvus* and *Homo sapiens* (Hela cells) with XbaI (Promega). The digests were run overnight in a 0.8% agarose gel and blotted onto a Hybond-N+ membrane (Amersham) according to the manufacturer's instructions. Blots were hybridized in PerfectHyb Plus hybridization buffer (Sigma) at 65°C either with a ∼1-kb fragment of the pSIVgml *env* domain or with a ∼300 bp fragment of the pSIVgml LTR. Membranes were washed in 2×/0.1% SDS or 0.1× SSC/0.1% SDS at 65°C (i.e., medium to high stringency).The two probes were generated by PCR using the Env-F/6061-R1 and LTR-F/LTR-R primers respectively (Table S1), and subsequently [α-^32^P]dCTP-labelled (Random Primed DNA Labeling Kit, Roche). See Table S4 for the voucher numbers of the tissue samples used in this study. A picture of the ethidium bromide stained gels used to prepare the blots is shown in Figure S2.

### Phylogenetic Analyses

Three sets of phylogenetic analyses were conducted. The first one aimed at assessing formally the phylogenetic relationships between pSIV and other retroviruses and was based on an alignment including the 150 most conserved amino acids of the reverse transcriptase domain extracted from of a set of various retroviruses. The second one aimed at evaluating the support for a putative sister relationship between pSIV and other described primate lentiviruses and was based on an alignment including the 2350 most conserved nucleotides of *gag*-*pol* of all lentiviruses for which whole genome sequence is available. We also conducted phylogenetic analyses of a number of LTRs sequenced in the various species of lemurs in order to test whether pSIV was endogenized once in the common ancestor of *Cheirogaleus*+*Microcebus* or twice independently on the *Cheirogaleus* and *Microcebus* lineages. Sequences were aligned by hand using BioEdit [34] and the alignments (available in Datasets S1, S2 and S3) were submitted to Bayesian and Maximum Likelihood analyses using MrBayes [35] and PHYML [36]. For both types of analyses, we used the GTR+I+G model for the nucleotide dataset, as suggested by the AIC criterion in MrModeltest [37] and the rtREV model [38] for the amino acid dataset. Bayesian analyses were run for 5 million generations with a sampling frequency of one tree/set of parameters every 100 generations. 12,500 trees were discarded as burn-in before summarizing the tree samples. Maximum Likelihood support was evaluated via nonparametric bootstrap analyses using 1000 pseudo replicates of the original matrix. Accession numbers of the sequences used together with the pSIV consensus to construct the alignments are listed in Table S2.

### Dating

Genetic distances between paralogous and orthologous pSIV copies were calculated in MEGA 4.1 [39] using the Jukes-Cantor correction. The bushbaby (*Otolemur garnetti*) is the closest species to Malagasy lemurs for which an estimate of neutral substitution rate is available. In this species, neutral rates were estimated to vary between 2.83×10^−9^ and 3.29×10^−9^ substitutions per site per year based on the analysis of several families of ancestral repeats [24]. We used the average of these values, i.e., 3×10^−9^ (SD = 0.2×10^−9^; n = 4).

## Supporting Information

Figure S1 PCR verification of the *Microcebus murinus* contigs containing fragments of putative full-length pSIVgml copies. (A) The LTR fragments contained in the ABDC01505939 and ABDC01454290 contigs are 100% identical (1) suggesting that they correspond to the same LTR flanking a full length pSIV insertion in 5′ (see also Figure 1). The contig ABDC01306160 (2) contains a putative 3′ LTR flanked by a TSD that differs from (1) (ATTAT vs. CCCCA), suggesting that the pSIVgml fragment contained in ABDC01306160 could correspond to a second full-length pSIVgml insertion. We designed one primer in the region 5′ of the LTR on the ABDC01454290 contig (fl5′flank: 5′-GAG TAC TTG AGC CAC ATC TGC), one primer in the region 3′ of the LTR on the ABDC01306160 contig (3′flank6160: 5′-GCA AGC TGT GMC ACA TTT ATT BGC), and one primer in the 3′ flanking region of the putative full-length element flanked by the CCCCA TSD (fl3′flank: 5′-CTG TAT TCC AAG CAC ACA GC). As this region is not available in the WGS database, we used the 5′ flanking region of the CCCCA LTR in contig ABDC01454290 and blasted it against the human genome. We identified the region containing the pSIVgml empty insertion site in human, and designed the primer 3′ of this region on the human sequence. (B) We used these primers in combination with two primers designed in *env* (EnvF and EnvF2, Table S1) and one primer designed in *gag* (gagR2: 5′-ACT AGC GTG TCT AGT GC) to verify the validity of the different contigs. A 912-bp fragment was obtained using GagR2/fl5′flank (lane 1), confirming that contigs ABDC01505939 and ABDC01454290 contain pieces of the same full length copy. No PCR product was obtained using either EnvF2 (lane 2) or EnvF (lane 2′) with 3′flank6160 showing that the LTR flanked by the ATTAT TSD is not part of a full length copy and is most likely a solo-LTR. PCR products of 2045-bp and 1174-bp were obtained using fl3′flank in combination with EnvF (lane 3) and EnvF2 (lane 3′) respectively, further confirming that the proviral insertion flanked by the CCCCA TSD is a full-length provirus. Comparison of the *env* region sequenced from these PCR products revealed 100% similarity with the *env* fragment contained in the ABDC01306160 contig. As illustrated in (C), these data suggest that the contig ABDC01306160 results from a missassembly between a solo-LTR flanked by an ATTAT TSD (trace 1562873896) and a trace containing an *env* fragment of the full-length insertion and a fragment of the 3′ LTR.
(0.37 MB TIF)

Figure S2 Picture of the ethidium bromide stained gels corresponding to the blots in figure 2A (A) and figure 2B (B).
(3.83 MB TIF)

Figure S3 PCR validation of the absence of pSIV in *Mirza coquereli*. The first nine lanes show PCR results for three different sets of primers anchored at different positions within the internal sequence of pSIV (Table S1). For all three primer sets, bands of expected size were obtained in *Cheirogaleus medius* and *Microcebus griseorufus* but none in *Mirza coquereli*. As a positive control for *M. coquereli*, we used the primers 6061F/6061R to amplify the empty site for one of the pSIV solo LTR (as shown in Figure 5). Together these results are consistent with the Southern blot hybridizations (Figure 2) and PCR screening of orthologous insertions (Figure 5), showing that pSIV is present in *Microcebus* and *Cheirogaleus*, but not in *Mirza*.
(1.69 MB TIF)

Figure S4 Detailed map of a consensus pSIV reconstructed using one copy of pSIVgml (gray mouse lemur) and several copies of pSIVfdl (fat-tail dwarf lemur). We follow [9],[12] for the annotation format. The alignment of the multiple clones and contigs is provided in Dataset S1. The consensus for the LTR region was build using all LTR sequences obtained in the various lemur species. The three large ORFs (*gag*, *pol*, *env*) and the boundaries of their different domains were identified by comparison with the HIV1-HXB2 sequence [40]. The two small ORFs (*orf1* and *orf2*) do not show significant homology to any known lentiviral accessory gene but we note that they are located at a comparable genomic position than *vif* and *tat*, i.e., between *pol* and *env*, and the predicted proteins are very similar in size to those encoded by these accessory genes in other primate lentiviruses (*vif* is 192 aa and *tat* is 86 aa in HIV1-HXB2). The C-terminal ORF contains an arginine-rich motif similar to the nuclear localization signal (NLS) found in *rev* [16]. In addition, it is terminated by a leucine rich region that could potentially contain a nuclear export signal (NES), also characteristic of *rev* and other nuclear transporters [15],[16]. The inverted repeats at the ends of both LTRs and the putative promoter (TATATAA) are underlined and bold. The slippery sequence (AAAAAAC) and the hairpin/loop motifs characteristic of the retroviral *gag/pol* frameshift are indicated in the region where the *gag* and *pol* frames overlap. The *gag/pol* frameshift was identified using the program KnotInFrame [42].
(0.09 MB PDF)

Figure S5 Phylogenetic tree of a selection of different families of retroviruses obtained after analysis of the ∼150 most conserved amino acid of the reverse transcriptase domain. Numbers at each node correspond to Bayesian posterior probabilities ≥0.95 / bootstrap ML values ≥80. Accession numbers of the sequences used in this analysis are listed in Table S2. The alignment used for the analyses is provided in Dataset S4.
(0.32 MB TIF)

Table S1 List of the primers used to amplify the pSIV fragments in the various Malagasy lemur species. The name of the clones is as in Figure 1.
(0.03 MB DOC)

Table S2 Accession numbers of the retroviral sequences used in the various phylogenetic analyses conducted in this study. *the RELIK sequence was copied from Katzourakis et al. (2007). **the pSIV sequence corresponds to the consensus reconstructed in this study (Figure S4).
(0.02 MB DOC)

Table S3 Corrected genetic distances between the four orthologous solo LTRs shared by the *Microcebus* species sequenced in this study. Values are given in number of substitution per site for each pairwise comparison in the following order: solo LTR from contig ABDC01306160/solo LTR from contig ABDC01159233/solo LTR from contig ABDC01361523/solo LTR from contig ABDC01457045. Dashes indicate that the comparison was not possible because the solo-LTR was absent in one or more species.
(0.01 MB DOC)

Table S4 Specimen voucher numbers of the taxa used in this study. All *Microcebus* species were provided by the Field Museum of Natural History (FMNH), Chicago, IL. The four other genera were provided by the Duke Lemur Center, Durham, NC.
(0.03 MB DOC)

Dataset S1 Alignment (in fasta format) of the various pSIV clones and contigs used to reconstruct the pSIV consensus. All clones were deposited in Genbank (see Materials and Methods for accession numbers). CME = *Cheirogaleus medius*. MMU = *Microcebus murinus*.
(0.12 MB TXT)

Dataset S2 Alignment (in fasta format) of ∼2350 most conserved nucleotides of the *gag-pol* region of all lentiviruses for which a complete genome is available. Ambiguous regions were removed. Accession numbers of the sequences are listed in Table S2.
(0.08 MB TXT)

Dataset S3 Alignment (in fasta format) of the LTR fragments sequenced in the various species of lemurs. Ambiguous regions were removed. Sequences were deposited in Genbank (see Materials and Methods for accession numbers). CME = *Cheirogaleus medius*. MMU = *Microcebus murinus*. MTA = *Microcebus tavaratra*. MMY = *Microcebus myoxinus*. MRA = *Microcebus ravelobensis*. MSA = *Microcebus sambiranensis*. MGR = *Microcebus griseorufus*.
(0.01 MB TXT)

Dataset S4 Alignment (in fasta format) of the ∼150 most conserved amino acids of the reverse transcriptase domain of a selection of various retroviruses. Ambiguous regions were removed. Accession numbers of the sequences are listed in Table S2.
(0.01 MB TXT)
