# Supplementary figures and images for: Parallel Germline Infiltration of a Lentivirus in Two Malagasy Lemurs
Source: PLoS Genet. 2009 Mar 20;5(3):e1000425. doi: 10.1371/journal.pgen.1000425 (PMC2651035; doi:10.1371/journal.pgen.1000425)

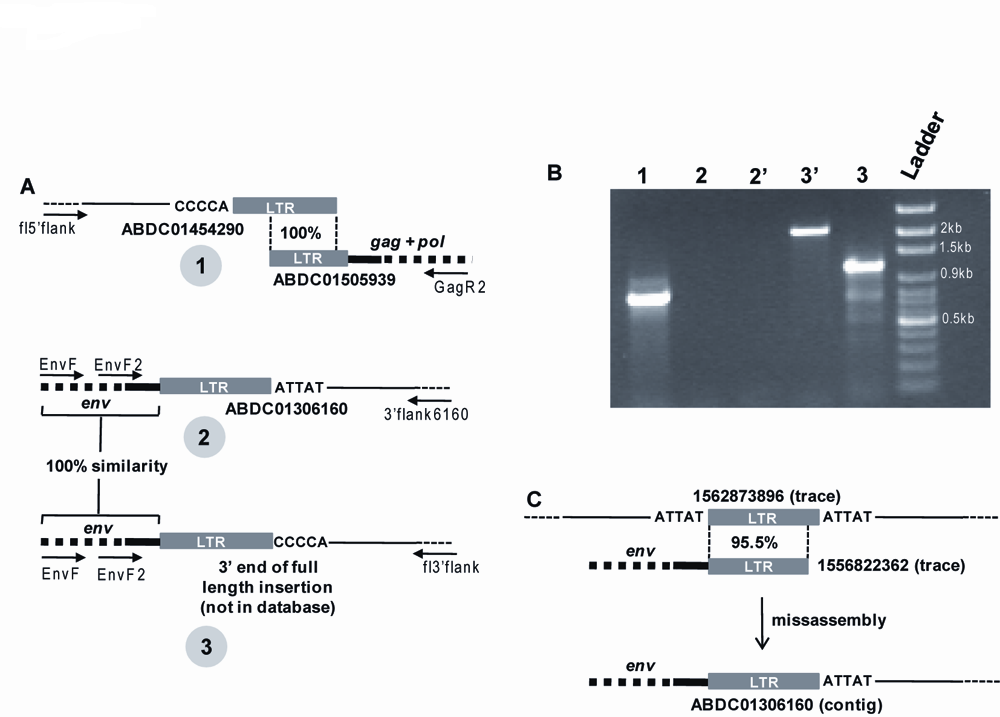

Supplement: Figure S1 — PCR verification of the Microcebus murinus contigs containing fragments of putative full-length pSIVgml copies. (A) The LTR fragments contained in the ABDC01505939 and ABDC01454290 contigs are 100% identical (1) suggesting that they correspond to the same LTR flanking a full length pSIV insertion in 5′ (see also Figure 1). The contig ABDC01306160 (2) contains a putative 3′ LTR flanked by a TSD that differs from (1) (ATTAT vs. CCCCA), suggesting that the pSIVgml fragment contained in ABDC01306160 could correspond to a second full-length pSIVgml insertion. We designed one primer in the region 5′ of the LTR on the ABDC01454290 contig (fl5′flank: 5′-GAG TAC TTG AGC CAC ATC TGC), one primer in the region 3′ of the LTR on the ABDC01306160 contig (3′flank6160: 5′-GCA AGC TGT GMC ACA TTT ATT BGC), and one primer in the 3′ flanking region of the putative full-length element flanked by the CCCCA TSD (fl3′flank: 5′-CTG TAT TCC AAG CAC ACA GC). As this region is not available in the WGS database, we used the 5′ flanking region of the CCCCA LTR in contig ABDC01454290 and blasted it against the human genome. We identified the region containing the pSIVgml empty insertion site in human, and designed the primer 3′ of this region on the human sequence. (B) We used these primers in combination with two primers designed in env (EnvF and EnvF2, Table S1) and one primer designed in gag (gagR2: 5′-ACT AGC GTG TCT AGT GC) to verify the validity of the different contigs. A 912-bp fragment was obtained using GagR2/fl5′flank (lane 1), confirming that contigs ABDC01505939 and ABDC01454290 contain pieces of the same full length copy. No PCR product was obtained using either EnvF2 (lane 2) or EnvF (lane 2′) with 3′flank6160 showing that the LTR flanked by the ATTAT TSD is not part of a full length copy and is most likely a solo-LTR. PCR products of 2045-bp and 1174-bp were obtained using fl3′flank in combination with EnvF (lane 3) and EnvF2 (lane 3′) respectively, further confirming that the pr [file pgen.1000425.s001.tif]

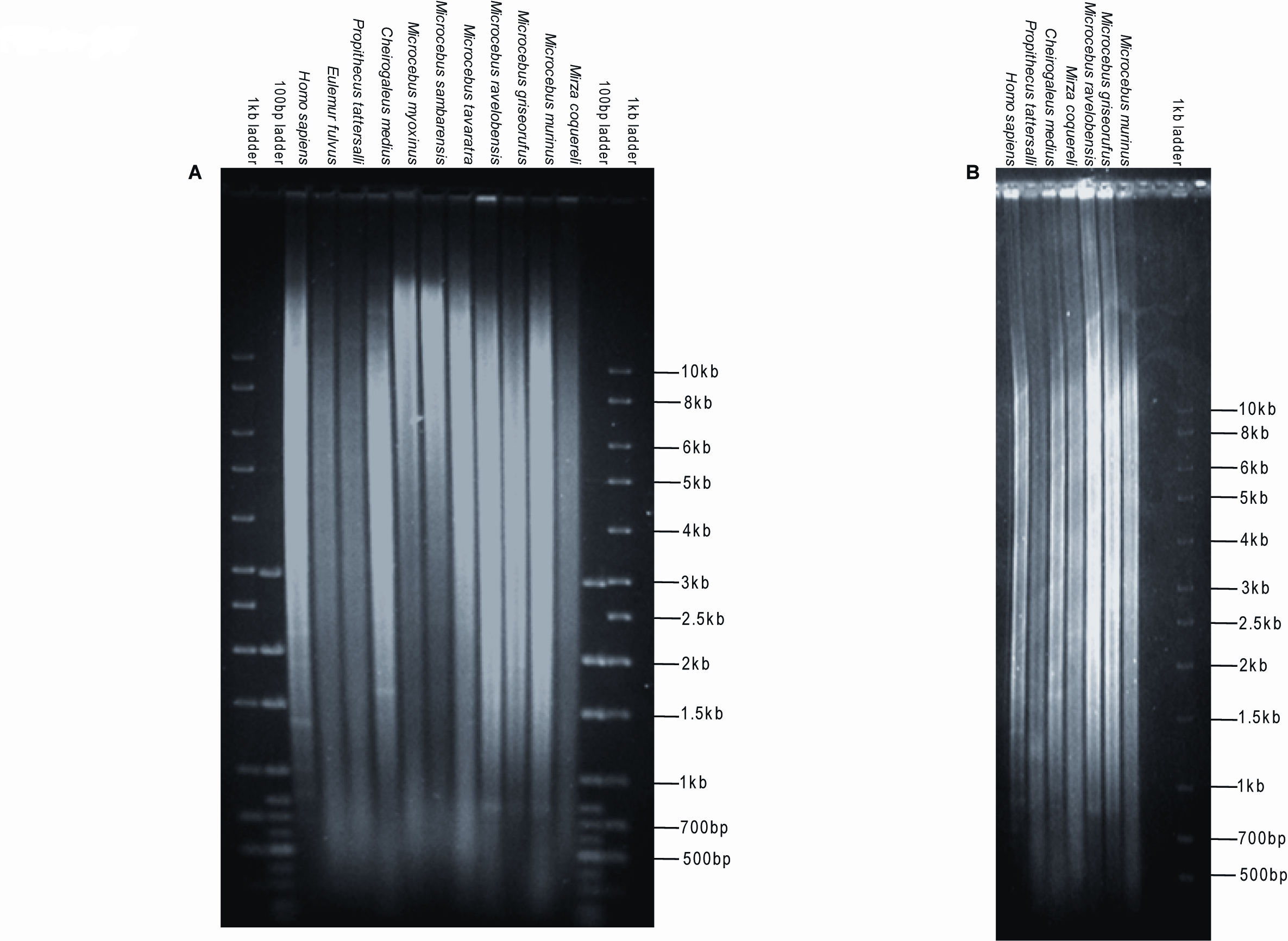

Supplement: Figure S2 — Picture of the ethidium bromide stained gels corresponding to the blots in figure 2A (A) and figure 2B (B). (3.83 MB TIF) [file pgen.1000425.s002.tif]

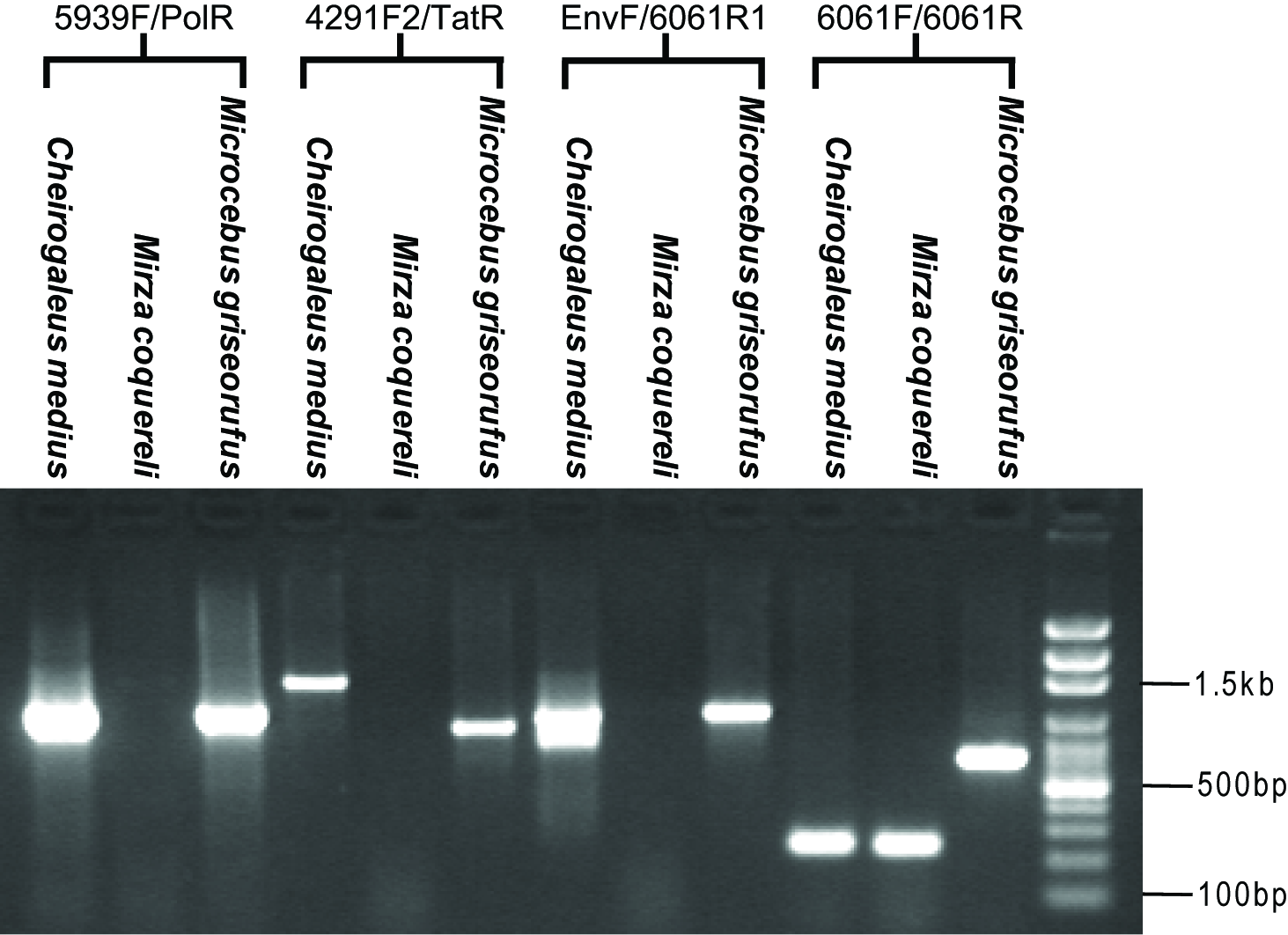

Supplement: Figure S3 — PCR validation of the absence of pSIV in Mirza coquereli. The first nine lanes show PCR results for three different sets of primers anchored at different positions within the internal sequence of pSIV (Table S1). For all three primer sets, bands of expected size were obtained in Cheirogaleus medius and Microcebus griseorufus but none in Mirza coquereli. As a positive control for M. coquereli, we used the primers 6061F/6061R to amplify the empty site for one of the pSIV solo LTR (as shown in Figure 5). Together these results are consistent with the Southern blot hybridizations (Figure 2) and PCR screening of orthologous insertions (Figure 5), showing that pSIV is present in Microcebus and Cheirogaleus, but not in Mirza. (1.69 MB TIF) [file pgen.1000425.s003.tif]

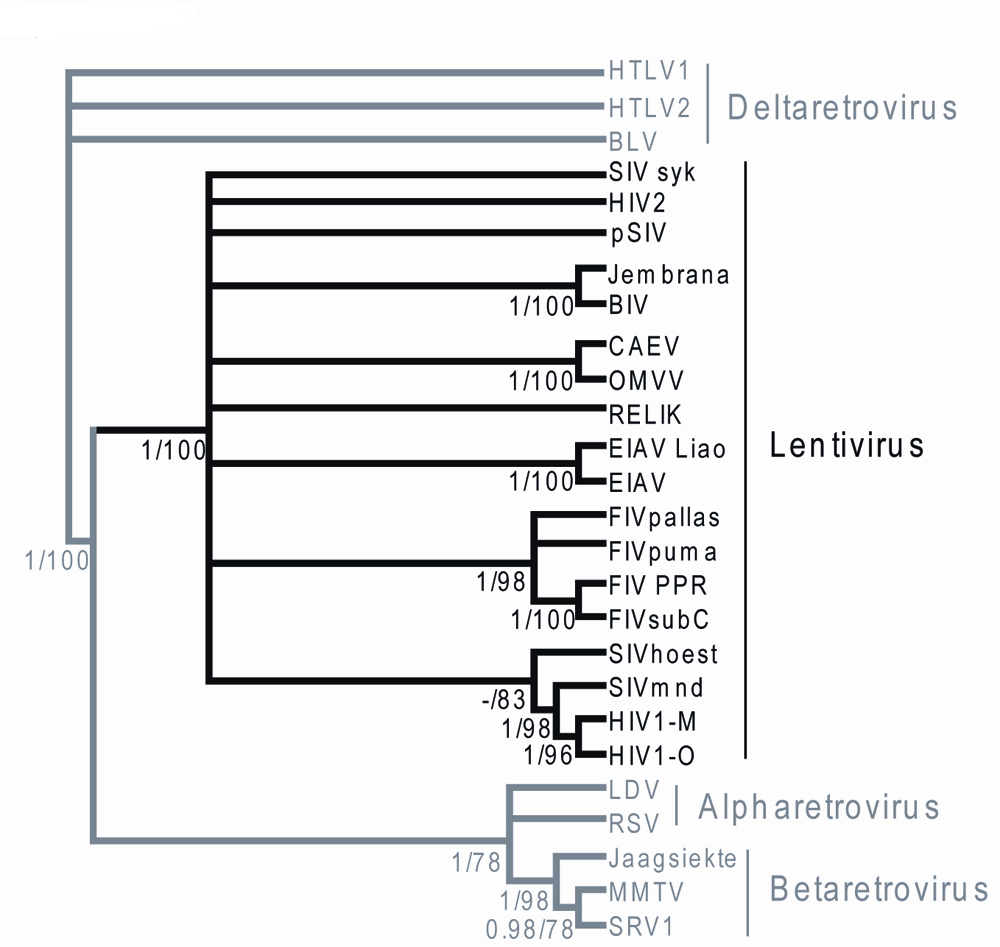

Supplement: Figure S5 — Phylogenetic tree of a selection of different families of retroviruses obtained after analysis of the ∼150 most conserved amino acid of the reverse transcriptase domain. Numbers at each node correspond to Bayesian posterior probabilities ≥0.95 / bootstrap ML values ≥80. Accession numbers of the sequences used in this analysis are listed in Table S2. The alignment used for the analyses is provided in Dataset S4. (0.32 MB TIF) [file pgen.1000425.s005.tif]
