# Supplementary material for: Parallel Germline Infiltration of a Lentivirus in Two Malagasy Lemurs
Source: PLoS Genet. 2009 Mar 20;5(3):e1000425. doi: 10.1371/journal.pgen.1000425 (PMC2651035; doi:10.1371/journal.pgen.1000425)
Supplement: Figure S4 — Detailed map of a consensus pSIV reconstructed using one copy of pSIVgml (gray mouse lemur) and several copies of pSIVfdl (fat-tail dwarf lemur). We follow [9],[12] for the annotation format. The alignment of the multiple clones and contigs is provided in Dataset S1. The consensus for the LTR region was build using all LTR sequences obtained in the various lemur species. The three large ORFs (gag, pol, env) and the boundaries of their different domains were identified by comparison with the HIV1-HXB2 sequence [40]. The two small ORFs (orf1 and orf2) do not show significant homology to any known lentiviral accessory gene but we note that they are located at a comparable genomic position than vif and tat, i.e., between pol and env, and the predicted proteins are very similar in size to those encoded by these accessory genes in other primate lentiviruses (vif is 192 aa and tat is 86 aa in HIV1-HXB2). The C-terminal ORF contains an arginine-rich motif similar to the nuclear localization signal (NLS) found in rev [16]. In addition, it is terminated by a leucine rich region that could potentially contain a nuclear export signal (NES), also characteristic of rev and other nuclear transporters [15],[16]. The inverted repeats at the ends of both LTRs and the putative promoter (TATATAA) are underlined and bold. The slippery sequence (AAAAAAC) and the hairpin/loop motifs characteristic of the retroviral gag/pol frameshift are indicated in the region where the gag and pol frames overlap. The gag/pol frameshift was identified using the program KnotInFrame [42]. (0.09 MB PDF) [file pgen.1000425.s004.pdf]

TGGAAGGAGATTTTGGGTGCAGCTGAGCACCCTAGTGACTGTGTGGGTGCAGCTGAGCACCCAGGTGACTGTGTACCAACGAGAGGTGCGAAAAGCCAAC 100

GAGAGGTGCGAAAGGTGCAGTGTGAGCATACAGGAAACCGCAACCGCAGACTGTCTCTCACACCTTTGCAGCTTACACAAGGACTTTGCTTTCTATTTGG 200

GGAAGGGGGGCTACTTCAGTACTTGGGGCTTAGGGAGGGCTTGGGGAGCA**TATATAA**GCCTGAGGTTGCCTAGCCTCGGGGCCCCCTCTCACATCTCTGG 300

GTCCGCCATCACCCAGACTCCAGAGTGTGGATCCACAATAAAGCTGTGCATCTTGACCCAGAGCTGTGTGTGTGTGCCGAGTCTTCTTGCCCTGGGGAAG 400

GCAACGCAAGTTGGCCCTTC**CA**GCTGGCGCCCGAACACGGGGCTTGACTTGACTTCTGCGAACGGTGAGTTTGTGGGAGTTGCAGGGCGAGATGGAGAAG 500
PBS
Gag> M E K
MA

GGGAAGATGGGTGATGGACAGTGTCTAGGGAAAGACACGGTGAAAAAGGCACAGCAGGTAAGAGTGAGAGGTACCGGGAAGCCCACGTACAAGTTAGGGA 600
G K M G D G Q C L G K D T V K K A Q Q V R V R G T G K P T Y K L G N

ACTTTGTCTGGGCAGTGAAGATAGCTGCAGCAGTAACAGGGGAGGACGCTCCTCGAAAATTAGAAGATCTATCAACAAAACAGCTGTGGGGTTTTTTAAA 700
F V W A V K I A A A V T G E D A P R K L E D L S T K Q L W G F L K

GGCGCTGGCCGCCAAAGCGGGGGCAGGGGACATTAATGTAGCACTAGACACCCTGCTAGTGTTATGGGTTACAGCAAAAGGGTTCACAGTGGAAAGTACA 800
A L A A K A G A G D I N V A L D T L L V L W V T A K G F T V E S T

GAGGGTGCCGCAGATGTGGCAAAGGTGGTAGGATGGCAAGAACAGCTCCATGGGAAGAAAGAGCAGGATCCGGAGGCAGAAGCAGCTGCCTACCCGGTTG 900
E G A A D V A K V V G W Q E Q L H G K K E Q D P E A E A A A Y P V V
MA CA

TAAATAGGGGACAGGGATGGGCTTATGAGCCTATGAGTACCAGAACTGTCGCAGCATGGATTCGCCAGACTGGAGAGAAGGGACTTACTAGTCCAGAAAC 1000
N R G Q G W A Y E P M S T R T V A A W I R Q T G E K G L T S P E T

AATCACATATTGGGGTTTAATATCCCAAGATCTGTCCAGTAGGGAACAGGTCCAACTGCTGGAAGTCGTTCCAGGACTTCAGGCAGACAAGGATATGCTG 1100
I T Y W G L I S Q D L S S R E Q V Q L L E V V P G L Q A D K D M L

GGGGCATATCTAGAAGAAAGGGCACGTGAGTGGGATGCGCAACCACAACAGCCATTGCCCTATACTTCTGCTCATATTAGGGGATTGACAGGGGATCAGG 1200
G A Y L E E R A R E W D A Q P Q Q P L P Y T S A H I R G L T G D Q A

CCTTTGCCATATCAGCGCAAGGGCGAGAAGCTGCGCAGGTCTTTAGGGCCTGGATAACGCAGGGCTTAATGAACTTGGCCCAGTTGCGGGCGCCACACCC 1300
F A I S A Q G R E A A Q V F R A W I T Q G L M N L A Q L R A P H P

AGGGTCAACAAAGATCTTGCAGGGACCAAAAGAGCCTTATGGGGAATTTATTAATAGACTGTTTCTCCAGATTAATCAGGAAGGAGCACCAGAGGAAGTA 1400
G S T K I L Q G P K E P Y G E F I N R L F L Q I N Q E G A P E E V

AAGACATATCTTAAGGGGCGTCTCAGCATTGAGAACGCTAATGCAGATTGCCAAAAGGCCATGAGCCATCTTAGGCCGGAAGACCCCTTGGAGGAGAAAA 1500
K T Y L K G R L S I E N A N A D C Q K A M S H L R P E D P L E E K I

TCAGAGCTTGTCAAAATGTAGGGAGTCAGTCTCACAAATTTGCAATGATGGCACAAATGTTTGGGGGGCAAAGAGGGGAAAAGGTGCAGGAGCAAAGGAA 1600
R A C Q N V G S Q S H K F A M M A Q M F G G Q R G E K V Q E Q R K
CA NC

GACACAGAAATGTTACAATTGTGGAAAATTTGGACATATGTCAAAGCAGTGCCGACAGTCCCACAAGTGTTATAACTGCGGGGAAGAGGGACATCTAGCA 1700
T Q K C Y N C G K F G H M S K Q C R Q S H K C Y N C G E E G H L A

"slippery sequence"
gag/pol frameshift/hairpin/loop
AAAAGTTGTCAAAGGCC**AAAAAAC**GGGAGGAGGAGCCCATCAGGGGCCCCACTAGAGATGACACCAGTGCAGCCTTCTGCACTGGAACCTCCCCAAGGGG 1800
Pol> K T G G G A H Q G P H * R * H Q C S L L H W N L P K G
K S C Q R P K N G R R S P S G A P L E M T P V Q P S A L E P P Q G G
PR

GAGGGAGATGGGGTTGTACCCCACCCTCAAGGAATTATTAAATTAGATCACCGGCCCCTGGTGACTCTGAAGGTAGGGGGACAATCGGTCACCCTCCTGA 1900
E G D G V V P H P Q G I I K L D H R P L V T L K V G G Q S V T L L I
G R W G C T P P S R N Y *
NC

TAGACACCGGGGCTGACAATACAATTATCCACCCTAAAGATTGGAAACCAGTAGGAATGGAAGAGGGGATAATTAACATTGGGGGAATTGGAGGTTCTCA 2000
D T G A D N T I I H P K D W K P V G M E E G I I N I G G I G G S Q

AAAGGGGGTATTATACAAACAAGTGCCTATTACTCTAGCAGATAGGCAGATACGGGGAACTGTCATAAGGGCAGTGACCCCTATAAATTTACTAGGGAGG 2100
K G V L Y K Q V P I T L A D R Q I R G T V I R A V T P I N L L G R

GACAATTTAGTATCACTAGGAATTGGAGTAGTGATGCTAATGGCACAGATGTCAGTAAAAATAGTGCCGCTGCCAGTTGAGTTAATGCCTGGCTGTGATG 2200
D N L V S L G I G V V M L M A Q M S V K I V P L P V E L M P G C D G

PR ↔ RT

GGCCAAGAGTAAAACAGTGGCCCCTTACGCAAGAGAAATATCAGGCTCTTGCTGAGATAGTATCTAAATTAGAAAAAGAGGGAAAAGTTAGTAGAGCAGA 2300
P R V K Q W P L T Q E K Y Q A L A E I V S K L E K E G K V S R A E

GGTAGGTAATCCCTACAACACTCCGGTGTTTGCCATTAAGAAAAAATCAGGCAAATGGAGAATGCTCATTGACTTTCGAGTGCTAAATGCTCGAACCAAA 2400
V G N P Y N T P V F A I K K K S G K W R M L I D F R V L N A R T K

AAGGGGGCTGAGTTTCAACTGGGCTTGCCCCACCCGGCAGGCCTACAAAAGAAAGATAATGTCACCATACTAGATATTGGTGATGCTTATTTTACCATAC 2500
K G A E F Q L G L P H P A G L Q K K D N V T I L D I G D A Y F T I P

CATTAGACCCCACATTTAAAAAGTACACTGCCTTTACTCTGATACCACCTAATAATCAGGGACCAGCCAGGAGATTTGTGTTTAATTGTCTACCACAAGG 2600
L D P T F K K Y T A F T L I P P N N Q G P A R R F V F N C L P Q G

GTGGGTGTGTAGTCCAGCCTTTTACCAAAGGACTATGAGTGACATCATACAGCCATGGAAACAAGCTCATCCTGAGGTCATGTTATATGTCTACATGGAT 2700
W V C S P A F Y Q R T M S D I I Q P W K Q A H P E V M L Y V Y M D

GACCTTCTAATCGGGACAGACCTCCCCTTGAGGGAGCATAGAAGGCTGGTCCAAGAGCTTAGGAGTATGCTTCTTGGCTGGGGCTTTGAAACTCCTGAAG 2800
D L L I G T D L P L R E H R R L V Q E L R S M L L G W G F E T P E E

AGAAAGTGCAGGACCAATGGCCACTACAGTGGATGGGGTATGAGTTACACCCTAATAAGTGGCAGTTGCAGGTCCGAAAATTAGAGTTACCAGATCACCC 2900
K V Q D Q W P L Q W M G Y E L H P N K W Q L Q V R K L E L P D H P

CACTTTAAATGAAGTCCAAAAACTGGTGGGAATTATTAATTGGGCTAGTCAAATTATATCAGGGCTTAAAATAAAGAAGCTTACTGCTATGATGGCAGGG 3000
T L N E V Q K L V G I I N W A S Q I I S G L K I K K L T A M M A G

AATCAGGATCTCAATAGAAAGATAGAATGGACTAAGGAAGCTAGAAAGGAAGCTGAAGAGGCAGCTAAGCTGCTCCAGGAGCTCCCAGCAGGGGGGTATG 3100
N Q D L N R K I E W T K E A R K E A E E A A K L L Q E L P A G G Y V

TCGACCCCTTGAAACAGGTGGAGGCTAGAATAGCTTTTGTAGGTTTCCGCGAGGTAGCCTACGACGTCCATCAGGAGAATATCATCCTTTGGTGTGGCAG 3200
D P L K Q V E A R I A F V G F R E V A Y D V H Q E N I I L W C G R

AGTAGGGTCCAGCAAAGCTCATTGTAATCCGGTGGACCTATGTGTAAAAGCAGCAATAAAAATAGGCAGGGAAACCTTGATTAGGCTAGGAGTGGTCCCC 3300
V G S S K A H C N P V D L C V K A A I K I G R E T L I R L G V V P

ATACTTACTGTCCCGGTAGCAAAAGATATCTGGGATAGTTTTGCATGGACTGCGGGGGAGGTAGCATGGTTCCCAGAAGTAAGACATCAGGCACCTCCTC 3400
I L T V P V A K D I W D S F A W T A G E V A W F P E V R H Q A P P L

TAATAGTTGAGAGATGGATGAAAATGGTGTCAGAACCCATTAAGGGGGCAAGAACTTGGTATATCGATGGATCTAAGAAACGGGGCCAAAAAGCTAGAGC 3500
I V E R W M K M V S E P I K G A R T W Y I D G S K K R G Q K A R A

RT ↔ RNaseH

AGGAATTTGGACAGAGGGAGAGAAGGCACAAGTACAGGAACTGGAGAGCTCAAATCAAAAAGCAGAATTGGCAGCCTTGTTGTATGCCTTACAGCAGGAA 3600
G I W T E G E K A Q V Q E L E S S N Q K A E L A A L L Y A L Q Q E

GACAAAGAATTAAACATTATCACTGATTCTCAATATGTAATGAAAGTGATGCGACGCGAGCTATGGGTCAGCGATTCTCCCTTGGTTCAGAGCATCATAC 3700
D K E L N I I T D S Q Y V M K V M R R E L W V S D S P L V Q S I I Q

AAGCAGCAGAGAAAAAACAGGCTATCTATTTAGATTGGGTGCCAGGTCATAAGGGAATCCCAGGAAATCAGAAGATTGATGAAGAAATTCAAAATTGGCA 3800

A A E K K Q A I Y L D W V P G H K G I P G N Q K I D E E I Q N W Q

AGGTTTGGTTATCCAAGGCACAGGTATCCTTCCTAAAAGAGAAGAGGATGTAGGCTATGATTTACAAATTCCAGAAGATGTGTACCTGCAGGGCTTAGAA 3900
G L V I Q G T G I L P K R E E D V G Y D L Q I P E D V Y L Q G L E
RNaseH ◄┴► dUTPase

AGGCGGTCCATTCCATTGAACTTGCGAGTTCAATGGGAAAAAGACCAATGGGGGTTGATTGTGGCAAAGTCCTCTATGGCTCAGATGGGGGTGATTCCTC 4000
R R S I P L N L R V Q W E K D Q W G L I V A K S S M A Q M G V I P L

TAGGTGGAGTCATAGATTCTGGATATAGAGGACCCATCATCCTCATCCTATGGAATCTTAATAGGAATGCAGTACTCCTTAAGGCCGGAAAAAGAGTGGC 4100
G G V I D S G Y R G P I I L I L W N L N R N A V L L K A G K R V A

TCAGCTAGTTATAATGTCTCTATTTCATGAGGAGTTGCAACAAGTTCAGCAGGTCAAAATTGACACGGCCCGAGGTGAAGGAGCATTTGGCTCCACTGGA 4200
Q L V I M S L F H E E L Q Q V Q Q V K I D T A R G E G A F G S T G

GCCTATTTCTTGGAGGCTATCCCTAGAGCAGAAAGTGATCATGAACTATGGCACTCGGGGGTTAAAGCTCTCATGCAGGATTTTGGAATGCCTCGCATGG 4300
A Y F L E A I P R A E S D H E L W H S G V K A L M Q D F G M P R M V
◄┴► IN
dUTPase

TGGCTAAAGCCATCGTGCAAAAATGTCCTGATTGCCAAGGGAAGGGATCAGCCATTACAGGGGTGGTGGATTACACCCCGGGGACATGGCAGATGGATGT 4400
A K A I V Q K C P D C Q G K G S A I T G V V D Y T P G T W Q M D V

TACCCACTGGGAAGGACATAAACTGTTAGTAGCACTTGAGACTGCTTCTGGGTTAACATGGGCTAAAATTATCCCTGATGAAACAGCCAAAACCACTTTG 4500
T H W E G H K L L V A L E T A S G L T W A K I I P D E T A K T T L

TTGGCTACATTAGAACTGCACAGTATTTTCAAAGTGAGTCACTTACATACAGATAATGGGCCTAATTTCACTGCTGAAAGATTTACTAATGCTCTTGCCT 4600
L A T L E L H S I F K V S H L H T D N G P N F T A E R F T N A L A W

GGTTAGGCATTAAGCACTCCACAGGTATCCCCTACAATCCTCAATCTCAAGGGGTAGTGGAATCTACCAATAAGTTGTTAAAAGAAATGCTCCACAAAAT 4700
L G I K H S T G I P Y N P Q S Q G V V E S T N K L L K E M L H K I

TAGACCCAAAATGGAGACAGTTCACGCGGCTGTCTATATGGCTTTATTTGCCATTAATTTTAAACAAAGGGGTGGAGTGGGAGGTACAACTAGATATGAA 4800
R P K M E T V H A A V Y M A L F A I N F K Q R G G V G G T T R Y E

AGACATTTAGACATGGGATTGGAAGACCTACAAAATTACCATTTCAAAAATTTGGACTCATACCATGTCTACTTTAAACAGCCACCTCAAAAAACCTGGC 4900
R H L D M G L E D L Q N Y H F K N L D S Y H V Y F K Q P P Q K T W Q

AGGGACCAGCTCGTCTCCTTTATAAGGGGCAGGGAGCGGTGGTCTGCGAGCATCAGGGAAAGACAATAGCAGTACCTAGACGCTACTGCAAGATCATAAC 5000
G P A R L L Y K G Q G A V V C E H Q G K T I A V P R R Y C K I I T

GGGAGTTTCTGATTCCCTCTCTGCAGATGGATAGCAGACAGAAATGGTACAACATCCCCGCTCCCCGGTGGCGCCCCTTTGGACGATGGGCTATCTCATT 5100
Orf1> M D S R Q K W Y N I P A P R W R P F G R W A I S L
G V S D S L S A D G *
IN ◄┘

GAGGCCATGGCCAGGAGAAGGGACCTATCAGCAAGTTAGAATATTGGGGCCACGAGATGAGTTCCTGATAATACATGGTGTACAAATTCACTCATCTGTG 5200
R P W P G E G T Y Q Q V R I L G P R D E F L I I H G V Q I H S S V

TTCTATGAGATTTGCCAACACAAGAACTGGGCACACTGTTTCTTGCATTGGTGGGGGCGTGGGAGGATTGCGCTAAGCTGGGGGGAGGCTGCTGCATGGG 5300
F Y E I C Q H K N W A H C F L H W W G R G R I A L S W G E A A A W E

AAAACTACAGCACCTTTCATACTTCGTGTAATTTAAAGGTCAAACATCATCTCCCGGTAGTGAACTTAGCCCTCAACCTACGGGCAGGAAATCTCAAGGG 5400
N Y S T F H T S C N L K V K H H L P V V N L A L N L R A G N L K G

ATTATGTGCAGTAGAGGCAATTGTCAACTTAGGGGAGCAAGGCTTTTATACGGAAGAGAACTTGCAAGAGCGAGTGCTGATGAGCATGGTCAGGGCCCAT 5500
L C A V E A I V N L G E Q G F Y T E E N L Q E R V L M S M V R A H

```
GAGCACCCTGGTAGCTGGGGAATAGCATACAGAATGCAGGAATGTTGGACTGACTCTGCTTTCCGAGTGTTTCAACAGCAGGGACCGTTGCTGCCGGAAG   5600
 E  H  P  G  S  W  G  I  A  Y  R  M  Q  E  C  W  T  D  S  A  F  R  V  F  Q  Q  Q  G  P  L  L  P  E  A

CATGCAACCAGATCGAGTTACCCTGAGGGATTGGAGTGAGTGGAGAGAGGCCTCTCTGCAGCTGTTGCCACGTATGGCACCAGTAACAGCGGAAGAAAAA   5700
Orf2>M  Q  P  D  R  V  T  L  R  D  W  S  E  W  R  E  A  S  L  Q  L  L  P  R  M  A  P  V  T  A  E  E  K
   C  N  Q  I  E  L  P  *

GACTGGTTCGACCTTCTAGCTCATGCTTTGCCTAAAGTACCCCTTTCTGTCTTAATGCTTATTTTCAGGGGGCCGCAAGAAAAATGGCGGGTAAGGTTGC   5800
 D  W  F  D  L  L  A  H  A  L  P  K  V  P  L  S  V  L  M  L  I  F  R  G  P  Q  E  K  W  R  V  R  L  Q
                                                                                    Env> M  A  G  K  V  A
                                                                                         SU

AACGCTGGCTTTGGTATACTCGCTTTGCGCAGGGGCACTGGGAACACAATTAGCATTGCTTAAAACCCCACCCATTGTTAGACTGCTTACCAATAATACA   5900
   R  W  L  W  Y  T  R  F  A  Q  G  H  W  E  H  N  *
  T  L  A  L  V  Y  S  L  C  A  G  A  L  G  T  Q  L  A  L  L  K  T  P  P  I  V  R  L  L  T  N  N  T

GAACCTCCTATAGTGTTCTGTGAGTCTGAGCAGGGGCACTTGGGATGTGCTCCAGCTTTATTTTCGTATGTTAATGCTTCTATCAATATATCTCACCCCT   6000
 E  P  P  I  V  F  C  E  S  E  Q  G  H  L  G  C  A  P  A  L  F  S  Y  V  N  A  S  I  N  I  S  H  P  L

TAGAGGAAGAGGTTGATCCATGGGATAGTTCTTTAGCATTGTTCACAGACTGGGTATCAGGAGCCCATATGCAATGGCTGACTCAGAGAGCCCAGGAATG   6100
   E  E  E  V  D  P  W  D  S  S  L  A  L  F  T  D  W  V  S  G  A  H  M  Q  W  L  T  Q  R  A  Q  E  W

GAGGGGATATTGCCAACCAATGAACTGTACGCAGGCTAATAACTTTACTAGAAATTGTACCAGGCCTTATGTGGATTATGAAGGTAGACCTGAAAACATT   6200
  R  G  Y  C  Q  P  M  N  C  T  Q  A  N  N  F  T  R  N  C  T  R  P  Y  V  D  Y  E  G  R  P  E  N  I

CAGGAGACAATTTCACAAATGCAGTTAAATTGTACTAATTCAACCTGTGTGAGGAAAGAGTGTAAACAAAGATTGTTCTTCCGGGGTAACCCACCTCTTG   6300
 Q  E  T  I  S  Q  M  Q  L  N  C  T  N  S  T  C  V  R  K  E  C  K  Q  R  L  F  F  R  G  N  P  P  L  D

ATGCTCAAACCTTTAGACTTTGTGTTAGACAACCTTTTGCTTTAAGAAGATGTCCACCAACCAATAGGATGGACTGGAGGCAACCTTATAATTGCTCTGA   6400
   A  Q  T  F  R  L  C  V  R  Q  P  F  A  L  R  R  C  P  P  T  N  R  M  D  W  R  Q  P  Y  N  C  S  E

GCAATGCCTAACTTCCTGTACAGAGGCAGTAAACGTAACTGTAGAAACTTTGTGGCAATCACAGGGAGTGTTAAACCCAAATCAAACAGGGGTGAGCTGT   6500
  Q  C  L  T  S  C  T  E  A  V  N  V  T  V  E  T  L  W  Q  S  Q  G  V  L  N  P  N  Q  T  G  V  S  C

TACGGAGAGGGGATGATGGTAACAGTCCAAACTGAACACGACCCCATTGGAATAAAGGTCTTGCAGACCATGAAAATACCAAAAATGACCTGTAATCTTA   6600
 Y  G  E  G  M  M  V  T  V  Q  T  E  H  D  P  I  G  I  K  V  L  Q  T  M  K  I  P  K  M  T  C  N  L  T

CAGGAGTTCAAAATAACAGTGGTCAAAAGGGCACAGTTGACCCCTGTTATTTCCTTTGCTATAATGCCACAAAGAAAGGAAGGGAAGGCAGAGGCAATCC   6700
   G  V  Q  N  N  S  G  Q  K  G  T  V  D  P  C  Y  F  L  C  Y  N  A  T  K  K  G  R  E  G  R  G  N  P

CATAGTGCTTATCTCTTGTAAGTACAATGGGACGTCTGGGACATTAACCAATTGTGAAAGAGTTTTTAAAGTGTCAATGCCAGGGCCACAAGATCCTCTA   6800
  I  V  L  I  S  C  K  Y  N  G  T  S  G  T  L  T  N  C  E  R  V  F  K  V  S  M  P  G  P  Q  D  P  L

TATTATCCAACCTATCCTAGGGAAAAGTGGTTACTGCATCTCCCAATGGAGGAAACAGGGGATCCCATACAATGTAATGCCTCTTTCCGGTGGCTACGTA   6900
 Y  Y  P  T  Y  P  R  E  K  W  L  L  H  L  P  M  E  E  T  G  D  P  I  Q  C  N  A  S  F  R  W  L  R  R

GGAGTGTAGCCTTACACGACACTGGGGTACAGGAGCCCGATATCAATAGCTCCTTTGGGGCAGAGGAAGCATGGAGAGATATGGTAGAAAGCTACATAGT   7000
   S  V  A  L  H  D  T  G  V  Q  E  P  D  I  N  S  S  F  G  A  E  E  A  W  R  D  M  V  E  S  Y  I  V

GTATCGTTTCCAGGAATGGACAGTGGTACCTGTGCAGGAGGGAGAAATAATATTATCTCGACCAAGGAGGGAACCGGTGACAATAATCTTGGCTCTGATA   7100
  Y  R  F  Q  E  W  T  V  V  P  V  Q  E  G  E  I  I  L  S  R  P  R  R  E  P  V  T  I  I  L  A  L  I
                                                                 SU      TM

TCGCTGTTCACTCTGGTTGGAACGGCTGTAAGTGTGGCCGCGATGCACCAGGTGGGAACTGTGGCGACCGCAGTTGGGGTACTCGTGGACAACGAAGGGC   7200
 S  L  F  T  L  V  G  T  A  V  S  V  A  A  M  H  Q  V  G  T  V  A  T  A  V  G  V  L  V  D  N  E  G  L
```

TCCTGTGGGAATCACAGAGGCGCTTGGGCAACTTAGTGGACCACCTAGCGTTCCAGGTACAGGTGTTACAAGCGAGGGTGCAGGCTATGGAGTATGTGTT 7300

L W E S Q R R L G N L V D H L A F Q V Q V L Q A R V Q A M E Y V L

GCAGGTTCAAGAATCTTGGTCTGAAATGGGATGTATTCCTTCACTGTGCCTTACGGACATTCCTTGGAACGATACATGGGGAGTTCCCAATATCACCCAG 7400

Q V Q E S W S E M G C I P S L C L T D I P W N D T W G V P N I T Q

ACTTGGAAAGAGTGGGATGATCAATTTTGGAAGAATTATGGTGAGGCTAGAGAGCTATGGCATAGACAGTTTTTAGAGGGACGAGGTAAATTGGGAAAGA 7500

T W K E W D D Q F W K N Y G E A R E L W H R Q F L E G R G K L G K I

TCCGCAGAGATTTAGCTCAAACTGCTCTAGCACAAAAAGTCAAGGATGCTCTCAATTCAATTCCTAAATGGTTGTGGACTGGCTTAGCAGTAATAATTGT 7600

R R D L A Q T A L A Q K V K D A L N S I P K W L W T G L A V I I V

ACTCATTATCATAATTCAAGTTGCTTGCATGGGGCTTAAAATGCTTACTAAAGCGGTTGTAAGTTGGGCAGGCTTTCTTGCATTGCAGCCAGAGGCGCCT 7700

L I I I I Q V A C M G L K M L T K A V V S W A G F L A L Q P E A P

Rev> S G C K L G R L S C I A A R G A S

CCCGACGTAGAGCCCGACGCCGCCGTCTGCGAAAACAACGGCTACGCAAATGGATGGCGCGACTACTGGCAACTCTGGAGCGGTGGGACCCTTTTCCAGA 7800

P D V E P D A A V C E N N G Y A N G W R D Y W Q L W S G G T L F Q M

R R R A R R R R L R K Q R L R K W M A R L L A T L E R W D P F P D

Arginine rich motif (putative NLS)

PPT

TGACTCGCCGGCCTTAAAAGAAAAAGAAAGGGTGGAC**TG**GAAGGAGATTTTGGGTGCAGCTGAGCACCCTAGTGACTGTGTGGGTGCAGCTGAGCACCCA 7900

D S P A L K E K E R V D W K E I L G A A E H P S D C V G A A E H P

T R R P *

GGTGACTGTGTACCAACGAGAGGTGCGAAAAGCCAACGAGAGGTGCGAAAGGTGCAGTGTGAGCATACAGGAAACCGCAACCGCAGACTGTCTCTCACAC 8000

G D C V P T R G A K S Q R E V R K V Q C E H T G N R N R R L S L T P

CTTTGCAGCTTACACAAGGACTTTGCTTTCTATTTGGGGAAGGGGGGCTACTTCAGTACTTGGGGCTTAGGGAGGGCTTGGGGAGCATATATAAGCCTGA 8100

L Q L T Q G L C F L F G E G G L L Q Y L G L R E G L G S I Y K P E

Leucine rich region (putative NES)

GGTTGCCTAGCCTCGGGGCCCCCTCTCACATCTCTGGGTCCGCCATCACCCAGACTCCAGAGTGTGGATCCACAATAAAGCTGTGCATCTTGACCCAGAG 8200

V A *

CTGTGTGTGTGTGCCGAGTCTTCTTGCCCTGGGGAAGGCAACGCAAGTTGGCCCTTC**CA** 8300

**NES**	Nuclear export signal
**NLS**	Nuclear localization signal
**RT**	Reverse transcriptase
**IN**	Integrase
**PBS**	Primer binding site
**PPT**	Polypurine tract
**CA**	Capsid
**MA**	Matrix
**NC**	Nucleocapsid
**TM**	Envelope transmembrane protein
**SU**	Envelope surface protein
