# Supplementary material for: Parallel Germline Infiltration of a Lentivirus in Two Malagasy Lemurs
Source: PLoS Genet. 2009 Mar 20;5(3):e1000425. doi: 10.1371/journal.pgen.1000425 (PMC2651035; doi:10.1371/journal.pgen.1000425)
Supplement: Table S1 — List of the primers used to amplify the pSIV fragments in the various Malagasy lemur species. The name of the clones is as in Figure 1. (0.03 MB DOC) [file pgen.1000425.s006.doc]

| **Name** | **Sequence** | **Position on Consensus LELV** | **Clones** |
| --- | --- | --- | --- |
| LTR R | 5'-TCTGGAGTCTGGGTGATGG-3' | 306-324 | LTR |
| LTR F | 5'-TAGTGACTGTGTGGGTGC-3' | 33-50 | CMEa, LTR |
| Gag R | 5'-GCCTTTGCCATATCAGCG-3' | 1200-1217 | CMEa |
| Gag F | 5'-CTCATATTAGGGGATTGACAGG-3' | 1171-1192 | CMEb |
| 4291 R | 5'-CAATGTATGGTAAAATAAGCATCACC-3' | 2479-2505 | CMEb, MMU1 |
| Pol R | 5'-TGTCCAAATTCCTGCTCTAGC-3' | 3493-3513 | CMEc |
| 5939 F | 5'-GGCTGAGTTTCAACTGGG-3' | 2406-2423 | CMEc, MMU1 |
| Pol F | 5'-GGGCAAGAACTTGGTATATCG-3' | 3446-3466 | CMEd |
| Pol R2 | 5'-ATAAAGCCATATAGACAGCCG-3' | 4727-4747 | CMEd |
| 4291 F2 | 5'-CCACAAAATTAGACCCAAAATGG-3' | 4692-4714 | CMEe, CMEf, MMU2 |
| Env R | 5'-GTACAGGAAGTTAGGCATTGC-3' | 6401-6421 | CMEe |
| Tat R | 5'-TCTAGTAAAGTTATTAGCCTGCG-3' | 6130-6152 | CMEf |
| Env F | 5'-TGTCCACCAACCAATAGGACG-3' | 6355-6371 | CMEg |
| 6061 R1 | 5'-CCCACTCCTTCCAAGTCTGG-3' | 7397-7416 | CMEg, MMU2 |
| 6061 R2 | 5'-AAAGACAGTCTGCGGTTGTGG-3' | 7980-8000 | CMEh |
| Env F2 | 5'-GGGCAACTTAGTGGAYCACC-3' | 7226-7245 | CMEh |
| Seq F | 5'-TATAGTGTTCTGTGAGTCTGAG-3' | 5909-5930 | MMU2 |
| Seq R | 5'-GTCCCATTGTACTTACAGGAG-3' | 6713-6733 | MMU2 |
