# Supplementary material for: Parallel Germline Infiltration of a Lentivirus in Two Malagasy Lemurs
Source: PLoS Genet. 2009 Mar 20;5(3):e1000425. doi: 10.1371/journal.pgen.1000425 (PMC2651035; doi:10.1371/journal.pgen.1000425)
Supplement: Table S2 — Accession numbers of the retroviral sequences used in the various phylogenetic analyses conducted in this study. *the RELIK sequence was copied from Katzourakis et al. (2007). **the pSIV sequence corresponds to the consensus reconstructed in this study (Figure S4). (0.02 MB DOC) [file pgen.1000425.s007.doc]

| RELIK | * |
| --- | --- |
| EIAV | NC_001450 |
| EIAVLiao | AF327877 |
| FIVsubC | AF474246 |
| FIVpallas | FIU56928 |
| FIVpuma | EF455614 |
| FIVPPR | FIVPPR |
| OMVV | NC_001511 |
| CAEV | NC_001463 |
| BIV | NC_001413 |
| Jembrana | NC_001654 |
| pSIV | ** |
| HIV1O | AY623602 |
| HIV2 | NC_001722 |
| HIV1M | EF029066 |
| SIVhoest | AF188116 |
| SIVmnd1 | AF328295 |
| SIVsyk | SIVCOMGNM |
| SIVagm | SIU58991 |
| SIVcol | AF301156 |
| SIVcpz | EF535994 |
| SIVdeb | AY523866 |
| SIVden | AJ580407 |
| SIVgsn | AF468659 |
| SIVmnd2 | NC_004455 |
| SIVmon | AY340701 |
| SIVmus | EF070331 |
| SIVsmm2 | AF334679 |
| SIVsmm1 | L09211 |
| SIVsun | AF131870 |
| SIVtal | AY655744 |
| LDV | X64337 |
| HTLV1 | P0C211 |
| BLV | P25059 |
| RSV | V01197 |
| SRV1 | SIVRV1CG |
| HTLV2 | Y13051 |
| Jaagsiekte | DQ838494 |
| MMTV | AF228552 |
