# Supplementary material for: Parallel Germline Infiltration of a Lentivirus in Two Malagasy Lemurs
Source: PLoS Genet. 2009 Mar 20;5(3):e1000425. doi: 10.1371/journal.pgen.1000425 (PMC2651035; doi:10.1371/journal.pgen.1000425)
Supplement: Table S3 — Corrected genetic distances between the four orthologous solo LTRs shared by the Microcebus species sequenced in this study. Values are given in number of substitution per site for each pairwise comparison in the following order: solo LTR from contig ABDC01306160/solo LTR from contig ABDC01159233/solo LTR from contig ABDC01361523/solo LTR from contig ABDC01457045. Dashes indicate that the comparison was not possible because the solo-LTR was absent in one or more species. (0.01 MB DOC) [file pgen.1000425.s008.doc]

|  | MGR | MTA | MMU |
| --- | --- | --- | --- |
| MTA | 0.021/-/-/- |  |  |
| MMU | 0.021/0.037/0.013/- | 0.034/-/-/- |  |
| MMY | 0.015/-/-/- | 0.026/-/-/- | 0.031/-/-/- |
| MRA | -/0.037/0.037/- | -/-/-/- | -/0.026/0.037/0.025 |
