# Supplementary material for: Parallel Germline Infiltration of a Lentivirus in Two Malagasy Lemurs
Source: PLoS Genet. 2009 Mar 20;5(3):e1000425. doi: 10.1371/journal.pgen.1000425 (PMC2651035; doi:10.1371/journal.pgen.1000425)
Supplement: Table S4 — Specimen voucher numbers of the taxa used in this study. All Microcebus species were provided by the Field Museum of Natural History (FMNH), Chicago, IL. The four other genera were provided by the Duke Lemur Center, Durham, NC. (0.03 MB DOC) [file pgen.1000425.s009.doc]

| *Microcebus murinus* | FMNH 161620 |
| --- | --- |
| *M. griseorufus* | FMNH 161639 |
| *M. ravelobensis* | FMNH 161616 |
| *M. tavaratra* | FMNH 161629 |
| *M. sambiranensis* | FMNH 161625 |
| *M. myoxinus* | FMNH 161644 |
| *Mirza coquereli* | 360m |
| *Cheirogaleus medius* | 1653f |
| *Propithecus tattersalli* | 6254f |
| *Eulemur fulvus* | 6287m |
